# Direct growth of crystalline triazine-based graphdiyne using surface-assisted deprotection–polymerisation†

**DOI:** 10.1039/d1sc03390e

**Published:** 2021-08-26

**Authors:** Ranjit Kulkarni, Jieyang Huang, Matthias Trunk, David Burmeister, Patrick Amsalem, Johannes Müller, Andréa Martin, Norbert Koch, Dustin Kass, Michael J. Bojdys

**Affiliations:** Humboldt-Universität zu Berlin, Department of Chemistry Brook-Taylor-Str. 2 12489 Berlin Germany m.j.bojdys.02@cantab.net; Humboldt-Universität zu Berlin, Institut für Physik and IRIS Adlershof Newtonstraße 15 12489 Berlin Germany; Department of Chemistry, King's College London, Britannia House Guy's Campus 7 Trinity Street London SE1 1DB UK

## Abstract

Graphdiyne polymers have interesting electronic properties due to their π-conjugated structure and modular composition. Most of the known synthetic pathways for graphdiyne polymers yield amorphous solids because the irreversible formation of carbon–carbon bonds proceeds under kinetic control and because of defects introduced by the inherent chemical lability of terminal alkyne bonds in the monomers. Here, we present a one-pot surface-assisted deprotection/polymerisation protocol for the synthesis of crystalline graphdiynes over a copper surface starting with stable trimethylsilylated alkyne monomers. In comparison to conventional polymerisation protocols, our method yields large-area crystalline thin graphdiyne films and, at the same time, minimises detrimental effects on the monomers like oxidation or cyclotrimerisation side reactions typically associated with terminal alkynes. A detailed study of the reaction mechanism reveals that the deprotection and polymerisation of the monomer is promoted by Cu(ii) oxide/hydroxide species on the as-received copper surface. These findings pave the way for the scalable synthesis of crystalline graphdiyne-based materials as cohesive thin films.

## Abstract 2

We present a one-pot deprotection/polymerisation protocol for the synthesis of crystalline graphdiynes on top of a copper surface starting with stable trimethylsilylated alkyne monomers.

## Graphical abstract

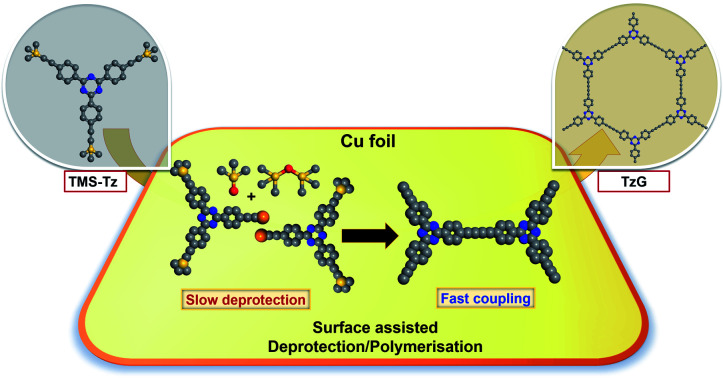

## Introduction

**Fig. 1:**
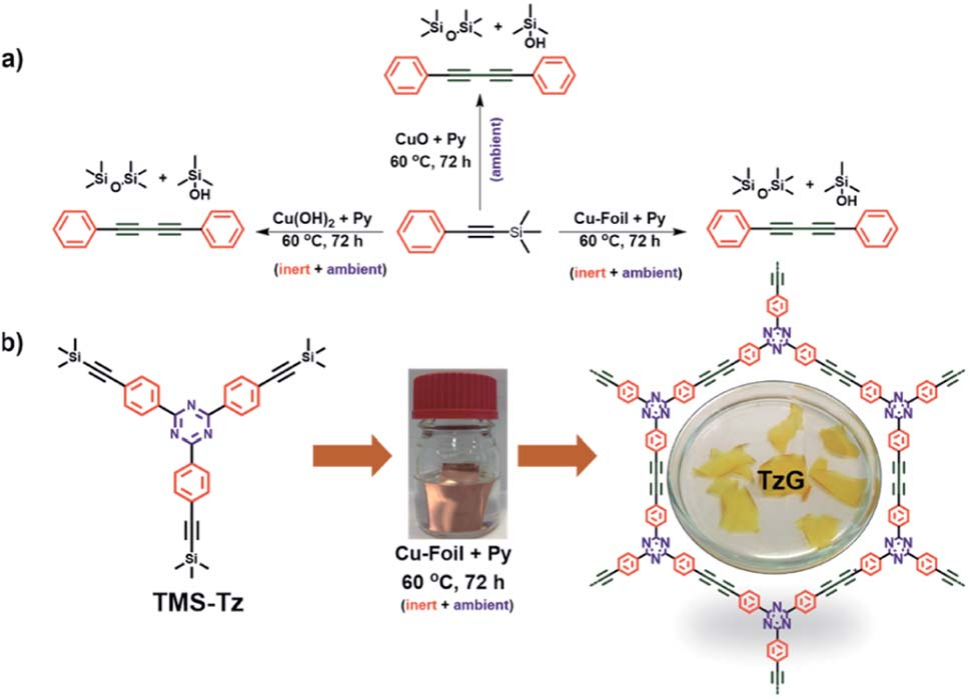
(a) Coupling of TMS–C≡C–Ph promoted by Cu(ii) (from CuO, Cu(OH)_2_, and on copper foil), and (b) synthetic route to a TzG polymer on copper foil.

Thin-layer, two-dimensional organic materials can complement the unique properties of graphene,^1^ because – unlike graphene – they offer the necessary synthetic modularity to achieve a non-zero bandgap. In recent years, a triazine (C_3_N_3_)-based, two-dimensional π-conjugated carbon nitride (TGCN) with a narrow band-gap was identified as a “beyond graphene” material.^2,3^ However, to this day TGCN remains an exotic and singular addition to the graphene family because its synthetic protocol requires high-temperature, high-pressure, and ionothermal conditions, and this type of linking chemistry does not readily translate to other, suitable molecular building blocks. Graphdiynes are a class of materials that satisfy two requirements for “post graphene” materials: (i) structurally, graphdiynes are two-dimensional, layered, and periodic,^4^ (ii) chemically, they are covalently linked, fully π-conjugated, and modular in their make-up.^5^ In a periodic graphdiyne polymer, all carbon atoms are sp^2^–sp hybridised. This enables charge-transport across the π-conjugated framework and is important in the development of future carbon-based electronic devices.^6–10^ Despite promising conceptual attributes, the synthesis of highly ordered, defect-free graphdiynes remains a challenge as most synthetic protocols yield amorphous powders.^11–15^ To address this, Glaser-type coupling, Eglinton coupling, and Glaser–Hay coupling have been proposed, where the oxidative homocoupling of terminal alkynes is achieved in the presence of dissolved copper salts or copper surface under alkaline conditions.^11,16,17^ However, the free rotation around aryl–alkyne single bond and detrimental oxidation and self-polymerization side reactions of the terminal alkynes have so far resulted in the formation of less ordered graphdiynes polymers.^6,9,18–20^ Additionally, the kinetics of the underlying coupling reactions determine the morphology of the graphdiyne polymer. We found that faster Sonogashira coupling yields amorphous graphdiyne polymers, while the more sluggish Glaser protocol leads to ordered, crystalline polymer frameworks.^21^ Alternatively, a modified Hiyama protocol has recently been introduced for the synthesis of graphdiyne polymers. Here, trimethylsilyl-protected alkynes are deprotected *in situ* and polymerise to graphdiynes. However such protocols use dissolved copper salts and or multi-component catalytic systems which adds to the chemical complexity which has so far limited the mechanistic understandings.^22,23^ Previously, we showed that monomers with terminal alkyne functional groups can be polymerised at the liquid–solid interface using a copper foil as a physical template and promoter of Glaser–Hay coupling and that this reaction yields continuous polymer films that grow on the copper surface. However, we found that during the initial stages of this reaction, the relatively high concentration of alkyne monomers at the copper interface favours a [2 + 2 + 2] cyclotrimerisation of surface-bound species.^6^ To address the shortcomings of conventional synthetic protocols for graphdiyne polymers, we propose to combine the Glaser and the modified Hiyama coupling. In the following, we obtain crystalline triazine-based graphdiyne (TzG) films directly from 2,4,6-tris(4-[(trimethylsilyl)ethynyl]phenyl)-1,3,5-triazine (TMS-Tz) monomers over a commercial copper foil surface. Where the copper surface (i) facilitates slow deprotection and fast coupling of the trimethylsilylated alkynes, (ii) acts as a template for the polymer growth which favors in-plane coupling of alkynes, and (iii) prevents the cyclotrimerization side-reactions typically associated with terminal alkynes. The resulting graphdiyne films have a thickness of up to 3 μm and lateral dimensions of 2–3 cm. In this study, we also shed light on the mechanism of monomer coupling. We observe that the copper oxide/hydroxide layer on as-received copper foil promotes simultaneous deprotection and polymerisation of trimethylsilylated alkynes (Fig. 1b). Our new lends itself well for the synthesis of large-scale, well-defined graphdiyne films in a flat, device-like set-up.

## Results and discussion

Inspired by the recently introduced, modified Hiyama coupling and our previous know-how in surface-assisted synthesis, we investigated the polymerisation of alkynyl silanes to graphdiyne polymers. In a control experiment, trimethylsilylethynylbenzene (TMS–C≡C–Ph) was dissolved in pyridine, and a commercial copper foil was immersed in the solution as a physical template and initiator of the coupling reaction. The reaction mixture was then stirred at 60 °C for 72 h, which afforded 1,4-diphenylbuta-1,3-diyne (Ph–C≡C–C≡C–Ph) as the only product in quantitative yields (Fig. 1a, for details, see ESI, Fig. S1–S3†). It is important to note that the reaction goes through an induction period, and no conversion was observed for the first 16 h monitored by thin-layer chromatography. This indicates that the coupling reaction proceeds *via* slow deprotection of the trimethylsilyl (TMS) groups and subsequent coupling to yield the diyne bridge, which was confirmed by gas chromatography (Fig. S4†). Further analysis of the reaction mixture was performed using liquid-state nuclear magnetic resonance, which revealed that the cleaved trimethyl silanes form silanols and silanes and do not interfere with product formation (Fig. S5–S8†). In a second control experiment without added copper foil, we observe no sign of deprotection or coupling of the trimethylsilylethynylbenzene (TMS–C≡C–Ph) molecule.

Based on these findings, we attempted to grow a triazine containing graphdiyne (TzG) on a copper surface directly using 2,4,6-tris(4-[(trimethylsilyl)ethynyl] phenyl)-1,3,5-triazine (TMS-Tz) monomers dissolved in pyridine, to gain control over the structure and morphology we optimized the reaction condition by varying the reaction temperature (Fig. S9–S12†). Similar to the control experiment, we did not observe any polymer growth in the initial 24 h of the experiment. Powder X-ray diffraction (PXRD) and Fourier-transform infrared spectroscopy (FTIR) screening of samples obtained at different reaction conditions show that TzG frameworks with the highest degree of order and full conversion of end groups are obtained at 60 °C (Fig. S10 and S11†). Free-standing flakes of TzG polymer are delaminated from the Cu foil and washed from copper residues embedded in the polymer matrix using a 1 M H_3_PO_4_ solution and repeated rinsing with solvents (for details see ESI, Fig. S12, and Video V1†).

**Fig. 2:**
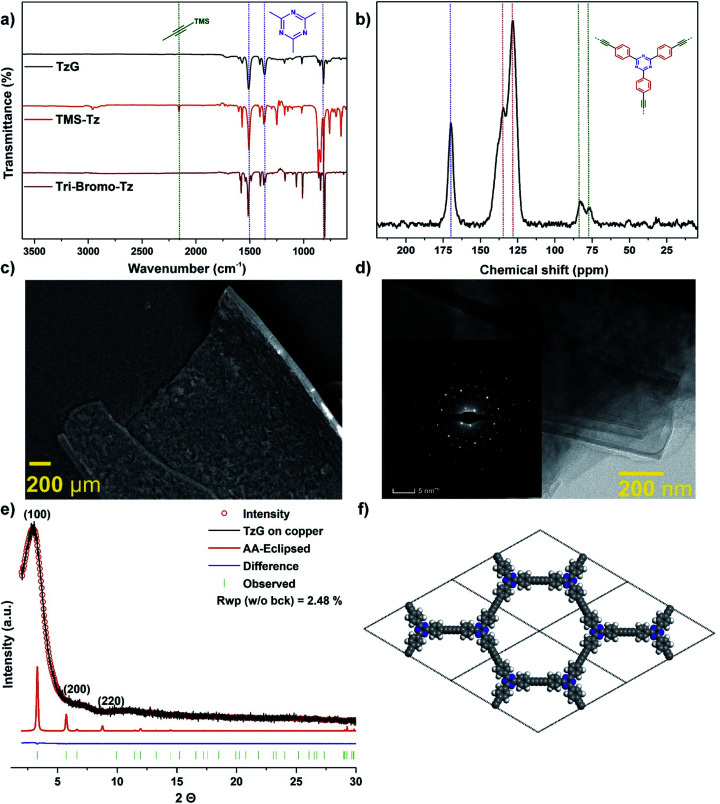
Composition and morphology of TzG polymers obtained *via* a coupling reaction on the copper surface using TMS-Tz monomer. (a) FTIR spectra of TzG (black) comparison with TMS-Tz (red) and tri-bromo–Tz monomers (maroon), (b) ^13^C solid-state CP-MAS NMR of TzG (black), the triazine carbon signal (blue dotted line) appears at 170 ppm, the quaternary diyne carbons (green dotted lines) at around ∼80 ppm and phenyl peaks (red dotted lines) between 120 and 140 ppm, (c) SEM images show layered structure with contours from the copper foil, (d) TEM images of TzG features highly crystalline sheets on the order of 500–1000 nm and corresponding electron diffraction images which shows pronounced hexagonal diffraction spots, (e) experimental (black line), Pawley refined (red circles), and predicted (thin red) PXRD patterns of TzG, Bragg peak positions (green), and the difference plot (experimental minus refined; blue) and (f) 2 × 2 unit cells (*a* = 30.78 Å, *c* = 3.06 Å) of the principal unit cell, carbon (grey), hydrogen (white), nitrogen (blue/violet), atoms are represented as spheres.

The chemical composition of isolated TzG flakes was confirmed by combustion elemental analysis after drying at 180 °C for 72 h as 83.50 wt%, 4.04 wt% (H), and 10.44 wt% (N), compared to theoretical values of 85.73 wt% (C), 3.17 wt% (H), and 11.11 wt% (N) (Table S1†). Thermogravimetric analysis (TGA) of the isolated TzG flakes under air shows a decomposition onset at approx. 400 °C and a residual mass of >0.6% at 800 °C (Fig. S13†). X-ray photoelectron spectroscopy (XPS) and energy-dispersive X-ray (EDX) analysis of TzG polymer flakes does not show any signs of silicon (from TMS endgroups) or copper environments (from atoms dislodged from the copper foil) (Fig. S14 and S15†). In addition, we found that the chemical etching with 1 M H_3_PO_4_ solution did not affect the chemical make-up of TzG. XPS data from the C 1s region shows characteristic signals of triazine carbons at 287.1 eV, sp carbons at 286.2 eV, and sp^2^ carbons at 284.4 eV, with integrated ratios of 1 : 2 : 6 that fit the composition of the TzG polymer.^6,24^ Further, XPS data from the N 1s region shows a peak corresponding to triazine nitrogen environments at 399.16 eV. Based on EDX analysis the C/N ratio was found to be 9 : 1, which matches the theoretical value. FTIR spectroscopy of TzG (Fig. 2a and S16†) shows no evidence of unreacted, deprotected alkyne C–H stretching bands which would be expected at 2970 cm^−1^ and 3300 cm^−1^. Importantly, the peaks corresponding to the stretching (at 1501 cm^−1^), breathing (at 1359 cm^−1^), and out-of-plane ring bending (at 816 cm^−1^) modes of the triazine ring are retained.^21,25,26^

The characteristic stretching mode of diyne (–C≡C–C≡C–) bridges was found at 2200 cm^−1^ by Raman spectroscopy (Fig. S17†).^6,21,27^ The chemical composition of the bulk TzG polymer was then analysed by ^13^C cross-polarization (CP) magic-angle spinning (MAS) solid-state nuclear magnetic resonance (NMR). We observe the presence of diyne bridges at 82 and 77 ppm. The triazine ring carbon environment is assigned to the peak at 170 ppm, and the phenyl carbon environments are assigned between 128–140 ppm (Fig. 2b).^6,21^

Powder X-ray diffraction data and electron diffraction pattern show the formation of a crystalline TzG framework (Fig. 2e and inset 2d). We modelled conceivable stacking modes using density functional theory. Several arrangements typical for TzG were considered: AA-1-inclined, AA-2-inclined, AA-1-serrated, AA-2-serrated, AA-eclipsed and ABC layering (Fig. S18 and S19†). Among the calculated modes, the AA-eclipsed packing mode gave the best match with the observed diffraction profile. The AA-eclipsed packing motif can be described by a hexagonal unit cell with parameters of *a* = 30.08 Å, *c* = 3.06 Å. This calculated AA stacking model was then used for the structure refinement using the Pawley method. TzG has a broad diffraction peak at 3.2° as well as smaller peaks at 6.6° and 11.3° at 2*θ* (Cu Kα *λ* = 1.5406 Å); these reflections correspond to the (100), (200), and (220) diffraction planes.

According to DFT calculations there is only a very low energy barrier (of up to 0.5 eV per unit cell) between the different stacking modes of TzG.^6,21^ Hence, there is a high likelihood of stacking defects and turbostratic disorder between neighbouring layers. As a consequence, pore channels are occluded and the sample appears to have no accessible surface area (by N_2_ BET at 77 K).

We further investigated the morphology of TzG using electron microscopy. Scanning electron microscopy (SEM) was used to study the morphology and layer thickness of the TzG (Fig. 2c and S20†). The isolated large films of TzG roll-up after drying, which indicates flexibility and stability towards physical stress.^28,29^ We estimated the thickness of TzG films from SEM images taken parallel to the basal plane of the layers as approx. 3 μm. The low scattering contrast and small irradiated sample volume of TzG films give rise to fairly poor information content in PXRD profiles. Thus, we performed high-resolution transmission electron microscopy (HR-TEM) and selected area electron diffraction (SAED) measurements to elucidate the microstructure of TzG films (Fig. 2d, S21 and S22†). The images of TzG revealed a uniform and continuous, layered structure with domain sizes exceeding 500 nm. SAED patterns are hexagonal and give a good match with the simulated SAED patterns of the AA-eclipsed structure with unit cell parameters of *a* = 30.78 Å, *c* = 3.06 Å.

The optical properties of the synthesized TzG films were then evaluated by solid-state UV/vis diffuse reflectance spectroscopy and solid-state photoluminescence (PL) spectroscopy. UV/vis spectra show that TzG has a discernible absorption edge at 480 nm (Fig. S23a†), corresponding to a direct optical band gap of 2.34 eV, and an indirect optical band gap of 1.76 eV according to the Kubelka–Munk function (Fig. S23b†). Solid-state PL spectra show an emission maximum at 511 nm (2.42 eV), which is closer to the calculated direct bandgap value (Fig. S24†), suggesting that the synthesized TzG is a direct bandgap semiconductor. All local microscopic and bulk analysis are consistent with the predicted properties of a diyne-bridge, ordered TzG framework. In the following, we will take a closer look at the mechanism of action underlying the synthesis of this material.

## Mechanistic investigation on the active species

**Fig. 3:**
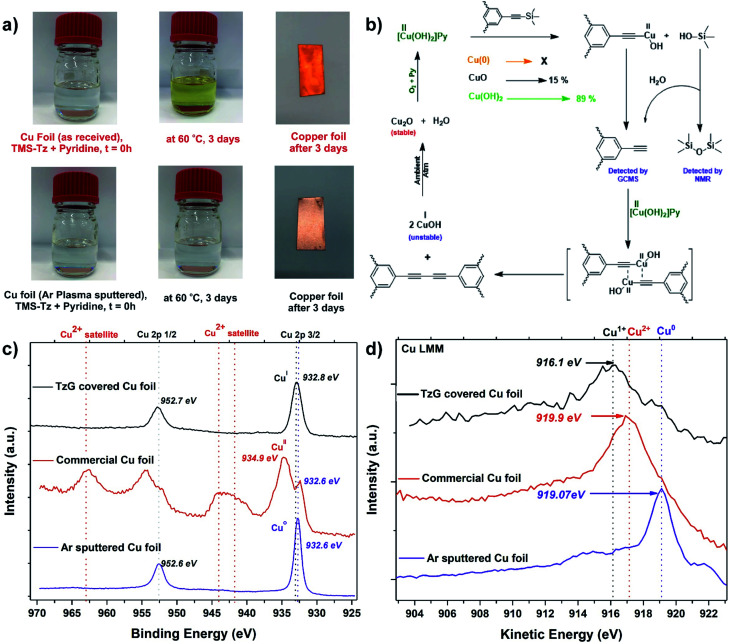
(a) Control experiments (1), comparison of polymerization attempts on an argon ion sputtered clean copper surface *vs.* as received copper foil, (b) plausible reaction mechanism for the copper-catalyzed deprotection/polymerization cascade, (c) high-resolution XPS (Cu 2p region) of argon ion sputtered clean copper surface (purple), as received copper surface (red), TzG covered copper surface (black) and (d) Auger Cu LMM spectra of argon ion sputtered clean copper surface (purple), as received copper surface (red) and TzG covered copper surface (black).

Conventional Glaser coupling of alkynes uses stoichiometric amounts of a copper(ii) species that are reductively eliminated as Cu(i) during the formation of the diyne-bridge. The re-oxidation of Cu(i) to Cu(ii) is then achieved by oxygen (Fig. 3b).^30^ Since the synthesis of TzG was performed under ambient atmospheric conditions, there is a possibility that co-dissolved oxygen plays a role in the proposed surface-assisted deprotection/polymerisation cascade of TMS-protected monomers. To confirm this, we performed the polymerisation with degassed pyridine in a glovebox environment and compared it with the experiments carried out without degassing of the solvent. In both reactions using degassed and not-degassed solvent, we observed polymer growth on the copper surfaces, which was then confirmed by FTIR spectroscopy (Fig. S25†). This indicates that the formation of TzG is facilitated by the copper(ii) species found on the surface of the as-received copper foil. Therefore, to better understand the role of surface species in the proposed deprotection/polymerisation cascade, we performed series of control experiments.

In control experiment (1) we prepared a clean Cu(0) surface by argon ion sputtering. XPS analysis confirmed the removal of any oxides present on the surface (Fig. 3c, S26†). No polymer growth was observed with the naked eye on the clean Cu(0) surface after heating at 60 °C for 72 h (Fig. 3a). In control experiment (2) we used CuO as a catalyst for the deprotection/coupling of model compound trimethylsilylethynylbenzene under inert and ambient conditions (Fig. S27 and S28†). These experiments revealed that CuO was only able to facilitate diyne formation under ambient atmospheric conditions. In control experiment (3) we performed the same model reaction under inert and ambient conditions using Cu(OH)_2_ as a catalyst (Fig. S27 and S28†). Here we observed that Cu(OH)_2_ readily facilitates the diyne formation under inert and ambient atmospheric conditions. In control experiment (4) we attempted to grow TzG using CuO under ambient atmospheric conditions (Fig. S29†), where we observed very little TzG growth (yield 15%). In control experiment (5) we attempted to grow TzG using Cu(OH)_2_ under identical conditions (Fig. S29†), which efficiently facilitated the formation of TzG (yield of 89%). From these experiments, we can conclude that the *in situ* deprotection/polymerisation cascade is predominantly caused by the surface hydroxide species present on the as-received copper foil. We propose a plausible reaction mechanism in that trimethylsilyl groups are deprotected on the copper surface and a copper(ii) acetylide complex is formed. This complex undergoes reductive elimination of Cu(i) during the formation of the butadiyne bridge. In this mechanism, trimethylsilanols (Me_3_Si–OH) observed *via* GCMS act as a proton source for the deprotected alkynes (Fig. 3b).

To further validate the proposed mechanism, we compared the oxidation states of the copper atoms on the foil's surface (i) in as-received state, (ii) after TzG growth, and (iii) after argon ion sputtering (Fig. 3c). The Cu 2p region in the XPS spectrum of the as-received copper foil shows the presence of Cu(ii) satellite signals between 940–945 eV and the main Cu 2p_3/2_ signal at 934.9 eV, unambiguously confirming the presence of the native Cu(ii) oxides.^31^ After TzG growth, the main Cu 2p_3/2_ component shifts to 932.8 eV which can either be assigned to Cu(0) or Cu(i) oxidation states.^31,32^ Thus, to differentiate between these two species, we compared the main Cu 2p_3/2_ component of the TzG covered Cu foil with the argon-ion sputtered copper foil, which showed a peak at a slightly lower energy of 932.6 eV. Since we encountered charging of TzG during XPS measurements, it is difficult to solely rely on the binding energies to determine the oxidation state of the probed element. Therefore, to elucidate the oxidation state of the copper foil after polymerisation, we measured the Cu LMM Auger region of the same copper surfaces (i) in the as-received state, (ii) after TzG growth, and (iii) after argon ion sputtering. The Auger electron spectra of the TzG coated copper foil show a broad and asymmetrical peak centered at 916.1 eV, indicating the presence of mainly Cu(i) species (Fig. 3d and S30†).^33,34^ The argon sputtered copper foil on the other hand shows a signal at 919.1 eV that can be unambiguously assigned to Cu(0). These findings substantiate the proposed mechanism (Fig. 3b).

## Conclusions

In summary, we present a simple, one-pot protocol for the synthesis of crystalline triazine-based graphdiyne films with a thickness of up to 3 μm and lateral dimensions of 2–3 cm directly from trimethylsilylated alkynes on commercial copper foil. In this work, we have merged the concepts of on-surface Glaser coupling with the ease-of-use of Hiyama coupling into a deprotection/polymerisation reaction. In principle, the here-presented method can be easily extended to a plethora of suitable monomer building blocks for the synthesis of freestanding, ordered, diyne-bridge polymer films.

## Data availability

The datasets supporting this article have been uploaded as part of the ESI.†

## Author contributions

All authors have actively contributed to the work presented in this paper R. K. and M. J. B. conceived the research project. R. K. planned and carried out the synthetic experiments, analysed the data and wrote the paper. J. H. carried out the XPS measurements and analysis. M. T. carried out the mechanistic studies and assisted in manuscript writing. D. B. carried out the argon plasma experiments and analysis. J. M. carried out the SEM and SEM-EDX measurements. P. A. performed the XPS measurements and Wagner plots. A. M. helped with the glove box experiments. M. D. performed the solid-state NMR experiments. D. K optimized the GC method and did the GCMS measurements and M. J. B. conceived the experiments helped in correcting the paper.

## Conflicts of interest

There are no conflicts to declare.

## Supplementary Material

SC-012-D1SC03390E-s001
