# Supplementary material for: Direct growth of crystalline triazine-based graphdiyne using surface-assisted deprotection–polymerisation
Source: Chem Sci. 2021 Aug 26;12(38):12661–6. doi: 10.1039/d1sc03390e (PMC8494036; doi:10.1039/d1sc03390e)
Supplement: SC-012-D1SC03390E-s001 [file SC-012-D1SC03390E-s001.pdf]

Electronic Supplementary Material (ESI) for Chemical Science.
This journal is © The Royal Society of Chemistry 2021

Supporting Information

Direct growth of crystalline triazine-based graphdiyne using surface-assisted deprotection-polymerisation

*Ranjit Kulkarni, [1] Jieyang Huang, [1] Matthias Trunk, [1] David Burmeister, [1] Patrick Amsalem, [2] Johannes Müller, [2] Andrea Martin, [1] Norbert Koch, [2] Dustin Kass, [1] and Michael J. Bojdys [1,3]**

Materials and Methods:

Materials:

Materials, unless otherwise stated, were commercially available and used without further purification. All the solvents used were of analytical grade and were used without further purification. 4-Bromobenzonitrile, trifluoromethanesulfonic acid (TFMSA), and triethylamine were purchased from Acros Organics. Pyridine from VWR. Copper foils from Goodfellow, 1-phenyl-2-trimethylsilylacetylene, $Pd(PPh_3)_4$, and CuI were purchased from Sigma-Aldrich.

Note: All the monomer synthesis was performed under inert conditions. All other control experiments and polymer synthesis were performed under ambient conditions or otherwise mentioned.

Elemental analysis (EA) (C, H, and N) analyses were performed using a PE 2400 Series II CHN Analyzer.

Fourier transformed infrared spectroscopy (FT-TR): spectra were recorded on an AVATAR 370 FT-IR spectrometer from Thermo Nicolet.

Thermogravimetric analysis (TGA): measurements were carried out under air or nitrogen atmosphere on a Mettler Toledo TGA STARe instrument with a heating rate of 10 K min^{-1}

Solid-state nuclear magnetic resonance (NMR) spectroscopy: The ^{13}C CP/MAS solid-state NMR spectra were obtained using a JEOL ECZ600R spectrometer. Samples were packed into 3.2 mm magic angle spinning rotors (MAS) and measurements were taken at MAS rates of 18 kHz. The ramped amplitude shape pulse was used during the cross-polarization. The contact time for CP was 5 ms. ^{1}H and ^{13}C NMR spectra of the monomers were recorded on a Bruker Advance 400 instrument. Chemical shifts (δ) are reported in ppm.

Solid-state (UV/Vis) and Fluorescence measurements: Solid-state diffuse reflectance Ultraviolet-visible spectroscopy (UV-Vis) spectra have been collected on Varian Cary 300 UV-Vis Spectrophotometer and solid-state fluorescence measurements recorded on Fluorolog FL3-22 fluorometer (Horiba-Jobin Yvon). The sample was excited at 400 nm, and emission spectra

were recorded in a suitable range centered around the emission maximum between 450 and 760 nm.

Solid-state Raman spectroscopy: Solid-state Raman spectra were recorded on a DXR Raman spectrometer (Thermo Scientific) interfaced with an Olympus microscope, employing a 20x objective. The 785 nm (diode-pumped solid-state laser) excitation lines were used. The laser power ranged from 0.1 to 1%. The full-scale grating was used for all measurements. The raw spectrum is reported due to the high fluorescence background.

X-ray photoelectron spectroscopy (XPS): XPS measurements were conducted using an Al Kα (1486.6 eV) source for excitation. The core level signals were fitted using Voigt peaks (Gaussian/Lorentzian) and a nonlinear Shirley-type background. The energy scale and binding energy of the spectrometer were calibrated with triazine species at the binding energies of the N 1s spectrum at 399.0 eV. For the XPS analysis of the Cu 2p region, The TzG on Copper was washed in a glove box to avoid surface oxidation, the sample manipulation for the measurements was also performed in a glove box environment. Further, the X-ray photoelectron spectroscopy (XPS) was performed in an ultrahigh vacuum chamber (base pressure $2x10^{-9}$ mbar) using a JEOL JPS-9030 set-up comprising a hemispherical photoelectron spectrometer and a monochromatic Al Kα (hν = 1486.6 eV) X-ray source. The Cu 2p and Cu LMM spectra were acquired with an overall energy resolution of 0.9 eV and 1.25 eV, respectively, as determined on a polycrystalline Ag 3d core level. The spectra of **TzG** on copper were corrected for charging by shifting the C1s peaks to 284.6 eV BE, to match the C1s binding energy of adventitious carbon observed on the as-received copper foil.

Powder X-ray (PXRD): measurements were performed with a Bruker D8 Advance diffractometer using Bruker AXS D8 Advanced SWAX diffractometer with Cu Kα (λ = 0.15406 nm) as a radiation source. Samples were measured from 1 to 60° 2θ with the step of 0.0102° 2θ secondary graphite monochromator and LYNXEYE XE detector.

Scanning electron microscopy (SEM) and Energy-dispersive X-ray spectroscopy (EDX): The sample morphology was investigated using a scanning electron microscope (SEM, GeminiSEM 500, Carl Zeiss GmbH, Germany) operating at 15kV. The samples were mounted on SEM stubs with adhesive carbon tape and imaged in plain view. Elemental mapping was carried out using the same SEM instrument equipped with an energy-dispersive X-ray spectrometer (EDX, XFlash 6130, Bruker GmbH, Germany) operating at 15kV.

Transmission electron microscopy (TEM): Transmission electron microscopy and the corresponding **selected area electron diffraction (SAED)** were carried out using an FEI TALOSTM F 200X, a 200 kV FEG operating at an accelerating voltage of 200 kV with a spherical aberration coefficient value of 5.6 nm. Images were recorded on CetaTM 16 M camera, which combined an embedded Piezo-enhanced stage.

Gas Chromatograph (GC): Gas chromatography: GC analysis was carried out by using an AGILENT 7890B gas chromatograph (HP5 column, 30 m) with a flame-ionization detector coupled to an EI-MS AGILENT 5977B spectrometer with a triple-axis detector. The instrument was equipped with an autoinjector Agilent G4513A (injection of approx. 10 µL). The GC method starts with an oven temperature of 50 ^{o}C (hold time 5 min) and includes two ramps (ramp 1: 50 ^{o}C to 190 ^{o}C, 10 ^{o}C /min; ramp 2: 190 ^{o}C to 300 ^{o}C, 20 ^{o}C /min), a total run-time of 24.5 min and a solvent delay of 2.9 min. MS peaks were analyzed and compared with the library database of NIST MS Search 2.3. All EI-MS spectra of the detected peaks were in good agreement with the library database of the expected substances.

Model reaction:

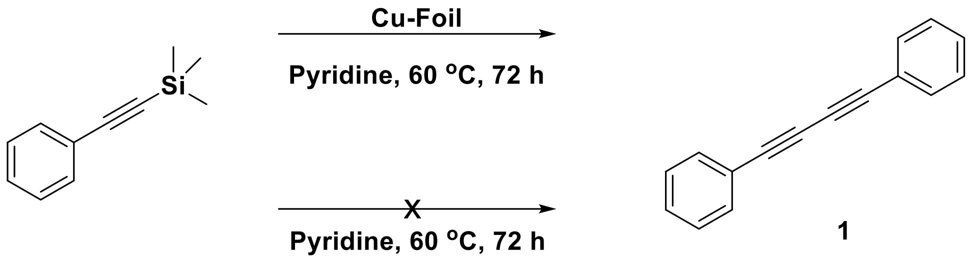

1

Fig. S1 Model reaction showing the efficacy of commercial copper foil as a coupling agent for a modified Hiyama-coupling.

Synthesis of 1,4-diphenylbuta-1,3-diyne (1)

In a round bottom flask containing 50 mL pyridine, trimethylsilylethynylbenzene (TMS-C≡C-Ph) (0.15 g, 0.84 mmol), was added. To this solution, small chunks of Cu foil 150 mg were added and the reaction mixture was heated at 60 °C for 72 h. After the reaction, the Cu chunks were physically removed and the reaction mixture was poured over water. Off-white solid precipitated out which was then collected by filtration and washed with water. The solid was then dried under vacuum for 12 h to yield (0.074 g, 0.36 mmol, 84 %) pale yellow solid of pure 1,4-diphenylbuta-1,3-diyne (Ph-C≡C-C≡C-Ph) **(1)**. ^{1}H NMR (400 MHz, $CDCl_3$): 7.55-7.52 (2H, M), 7.40-7.32 (3H, M). ^{13}C NMR (100 MHz, $CDCl_3$) δ 132.51, 129.22, 128.45, 121.80, 81.55, 73.91. A similar reaction without the Cu foil did not yield any product and the starting material remained unreacted observed by TLC. It is important to note that no conversion was observed for the first 16 h of the reaction.

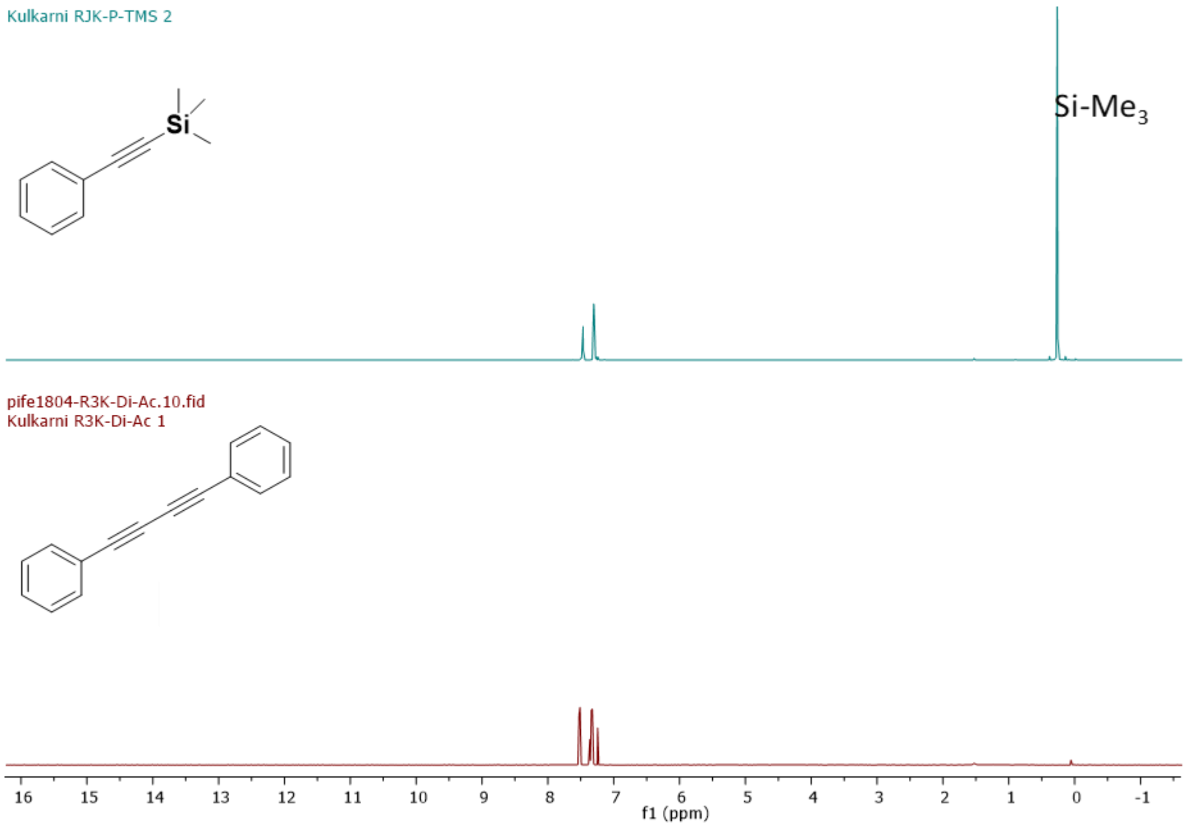

Fig. S2 ^{1}H NMR of the conversion of (TMS-C≡C-Ph) to diyne (Ph-C≡C-C≡C-Ph).

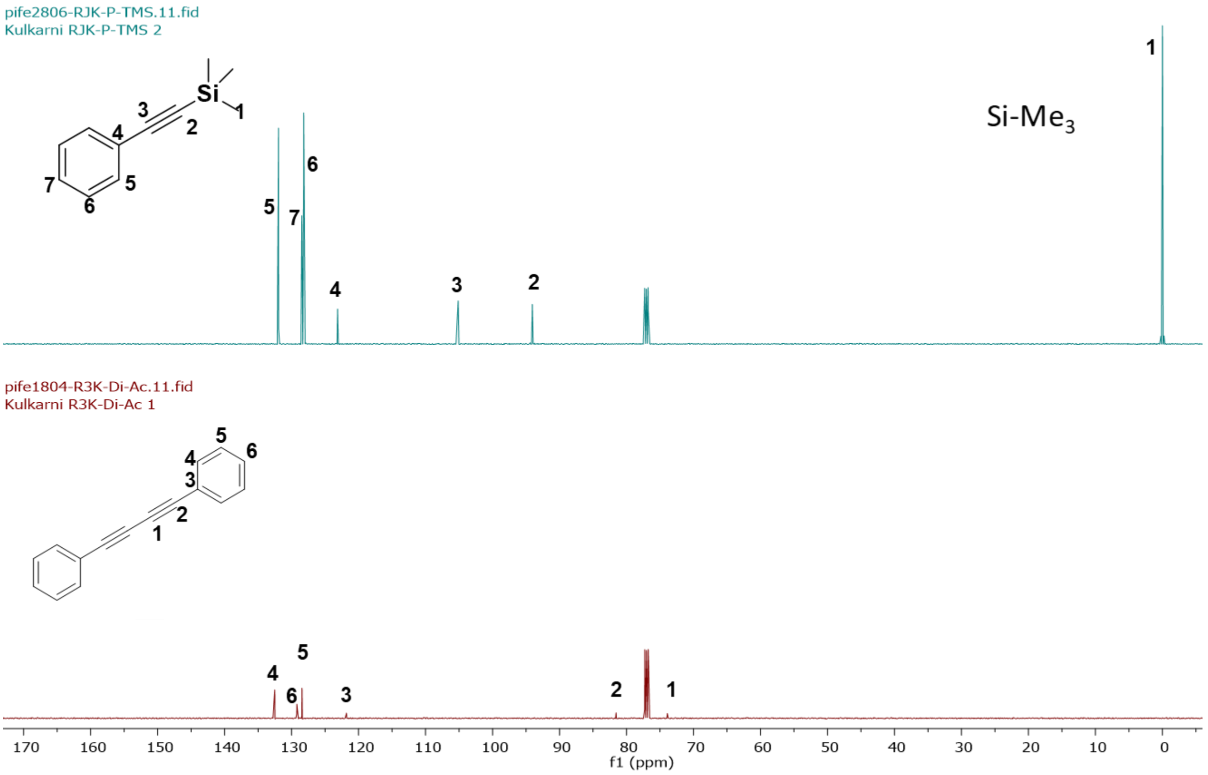

Fig. S3 ^{13}C NMR of the conversion of (TMS-C≡C-Ph) to diyne (Ph-C≡C-C≡C-Ph).

Gas chromatography:

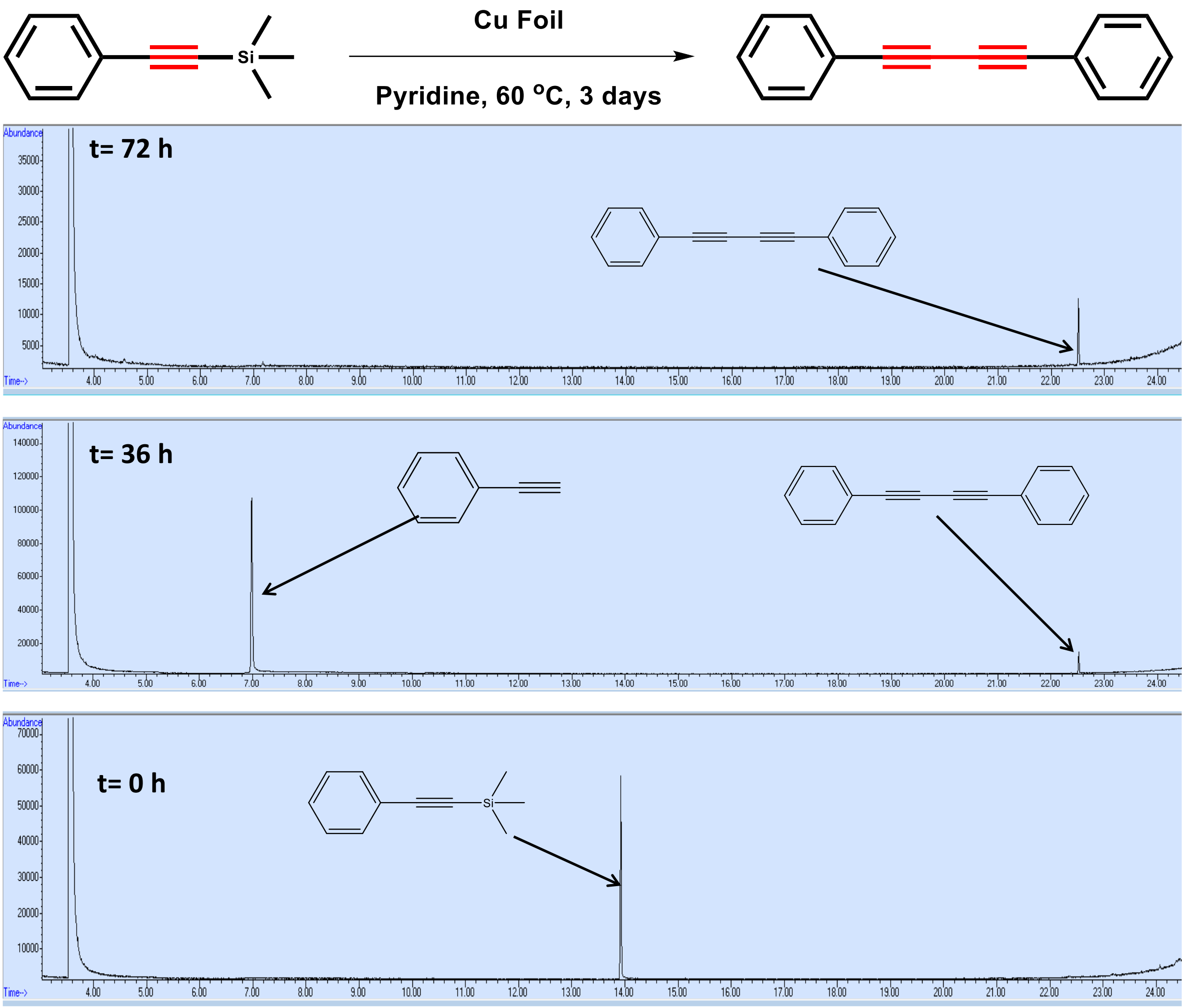

Fig. S4 Reaction monitoring by GCMS and different time intervals showing the efficacy of commercial copper foil as a coupling agent for a modified Hiyama-coupling. Sample preparation, was done by collecting the reaction mixtures at different time intervals, these samples were then extracted by partitioning between using $CHCl_3/H_2O$, finally, the organic layer was dried over anhydrous Na_2SO_4 and used for GCMS measurement (assignment of the visible peaks was done by comparison of the EI-MS spectra with the library database of NIST MS Search 2.3).

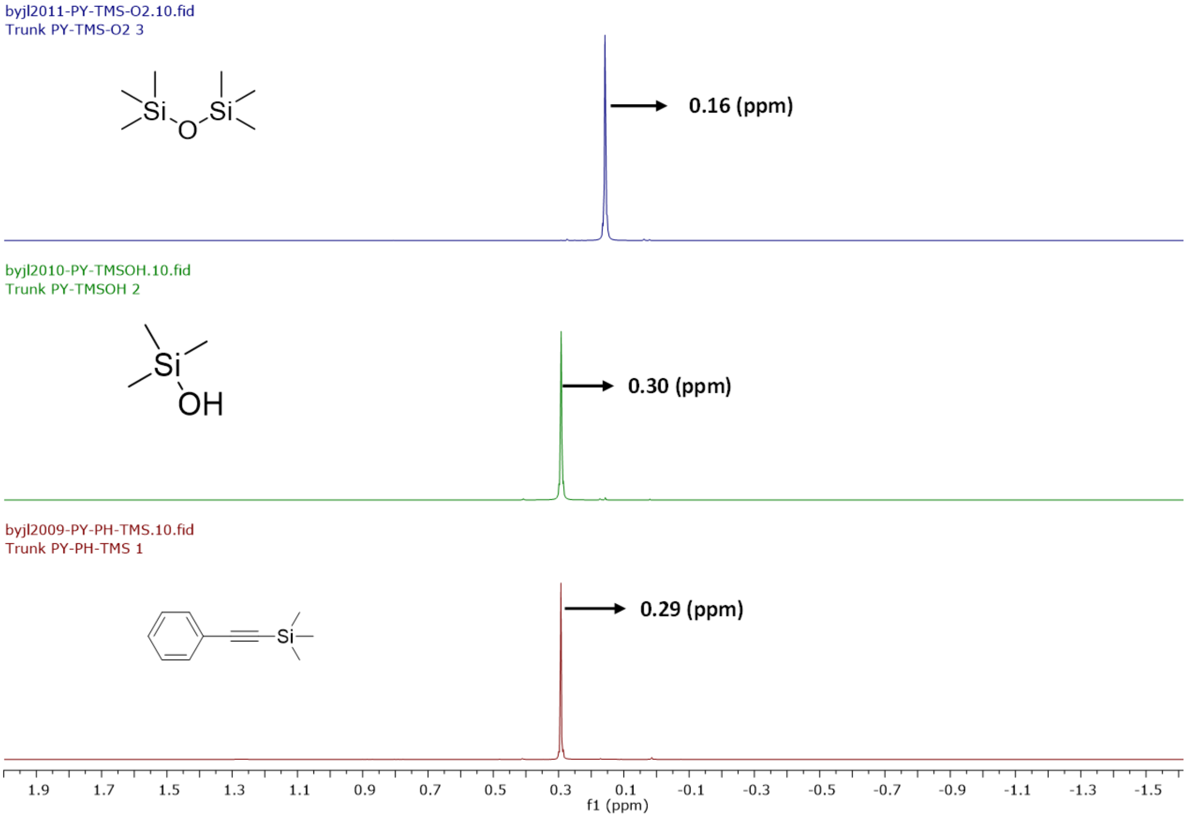

Fig. S5 ^{1}H NMR of trimethylsilylethynylbenzene (TMS-C≡C-Ph), and possible byproducts Trimethylsilanol and Hexamethyldisiloxane.

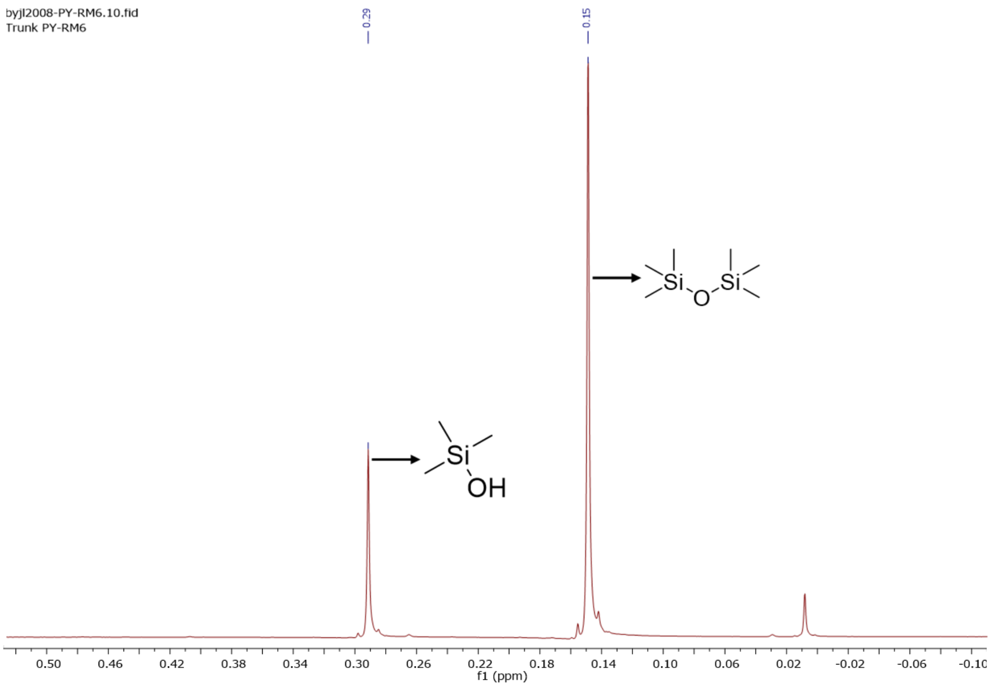

Fig. S6 ^{1}H NMR of the reaction mixture after 72 h confirming the formation of silanols, complete conversion of trimethylsilylethynylbenzene (TMS-C≡C-Ph) was confirmed by GCMS.

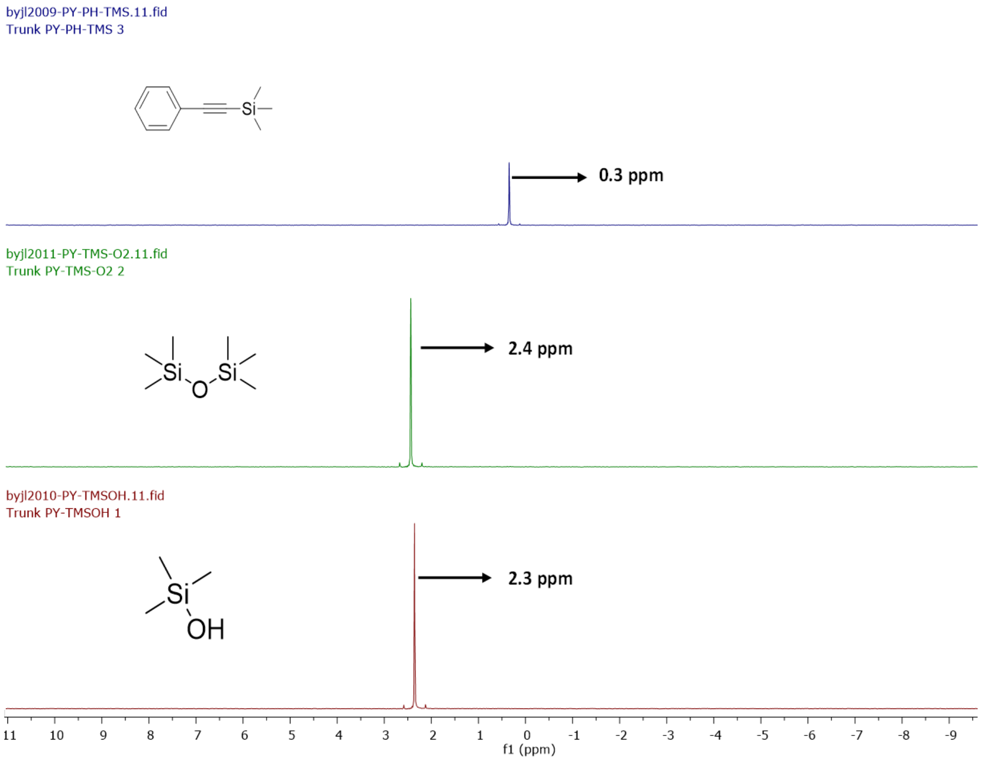

Fig. S7 ^{13}C NMR of trimethylsilylethynylbenzene (TMS-C≡C-Ph), and possible byproducts Trimethylsilanol and Hexamethyldisiloxane.

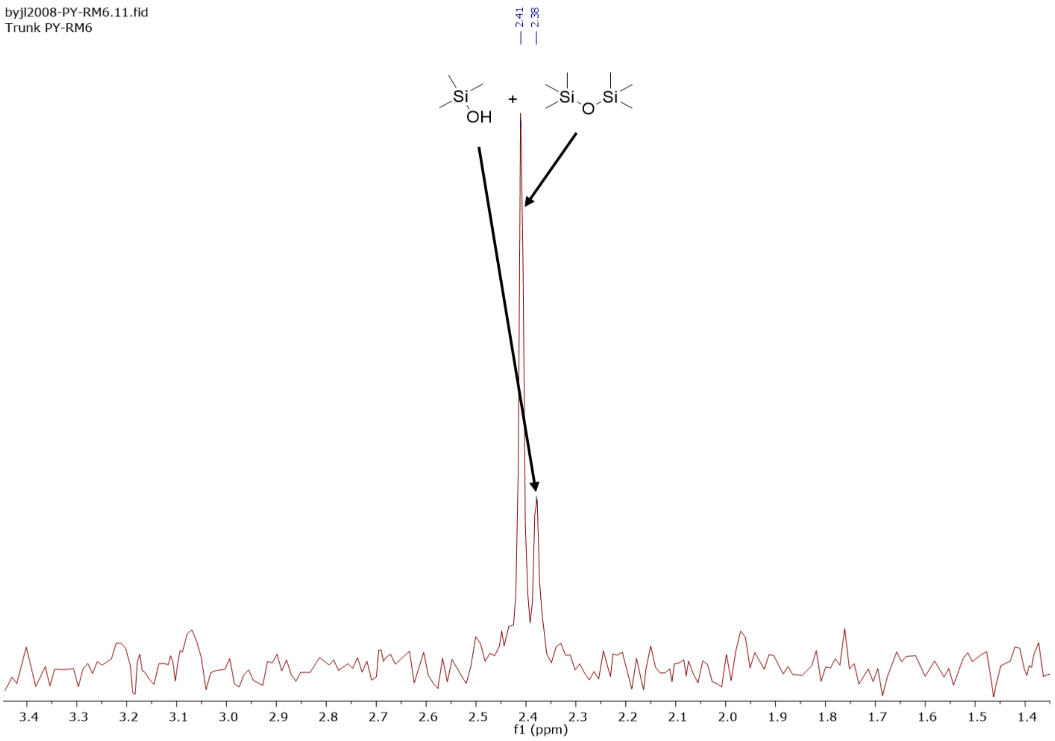

Fig. S8 ^{13}C NMR of the reaction mixture after 72 h confirming the formation of silanols, complete conversion of trimethylsilylethynylbenzene (TMS-C≡C-Ph) was confirmed by GCMS (Fig. S4).

Monomer synthesis:

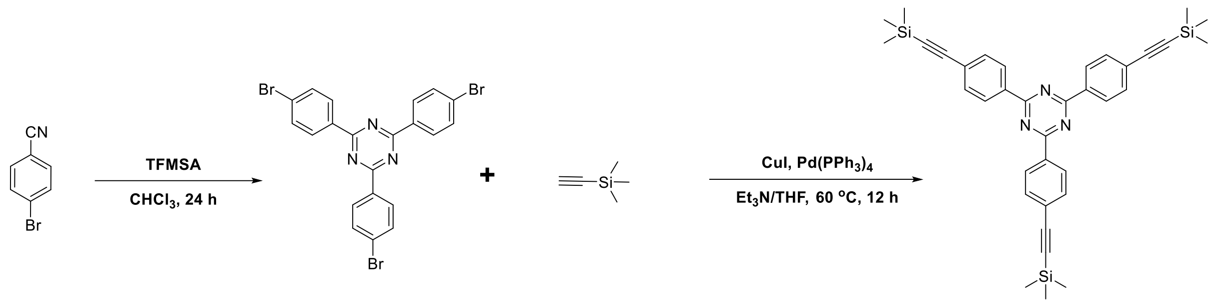

2 3

Fig. S9 Synthetic route for the synthesis 2,4,6-tris(4-bromophenyl)-1,3,5-triazine (**2**) and 2,4,6-tris(4-[(trimethylsilyl)ethynyl]phenyl)-1,3,5-triazine **(TMS-Tz)** (**3**).

Synthesis of 2,4,6-tris(4-bromophenyl)-1,3,5-triazine (2)

2,4,6-Tris(4-bromophenyl)-1,3,5-triazine was prepared from 4-bromobenzonitrile according to the method reported in our previous publication.[1] ^{1}H NMR (400 MHz, $CDCl_3$): δ = 8.61 (6H, d, J = 8.7 Hz, C_6H_4), 7.71 (6H, d, J = 8.7 Hz, C_6H_4).

Synthesis of 2,4,6-tris(4-[(trimethylsilyl)ethynyl]phenyl)-1,3,5-triazine (TMS-Tz) (3)

A 250 mL two-necked flask equipped with reflux condenser and a rubber septum was charged with 2,4,6-tris(4-bromophenyl)-1,3,5-triazine (2.0 g, 3.7 mmol), copper(I) iodide (0.19 g, 1.0 mmol) and tetrakis(triphenylphosphine)palladium(0) (0.58 g, 0.50 mmol) under argon. In a separate flask, Et_3N/THF (4:1 v/v, 50 mL) was degassed by argon purging for 30 min and then added through the septum to the flask containing the solids. Trimethylsilylacetylene (4.3 mL, 31 mmol) was then added to the mixture under Argon then stirred vigorously under Argon for 12 h at 60 °C. The reaction progress was monitored by TLC. After completion, the reaction mixture was filtered over celite. The mother liquor was then evaporated to dryness. The off-white solid obtained was then dissolved in CH_2Cl_2 and reprecipitated by adding methanol. The solid was then filtered and washed with copious amounts of MeOH and dried to yield pure product 2.1 g (95 %) of 2,4,6-tris(4-[(trimethylsilyl)ethynyl]phenyl)-1,3,5-triazine **(TzG), (2)** as a white solid. Spectral data was identical to the previous report.[2] ^{1}H NMR (400 MHz, $CDCl_3$): δ = 8.68 (6H, d, J = 8.4 Hz, C_6H_4), 7.65 (6H, d, J = 8.4 Hz, C_6H_4), 0.31 (27H, s, $Si(CH_3)_3$)

Trials for the synthesis of TzG Polymer:

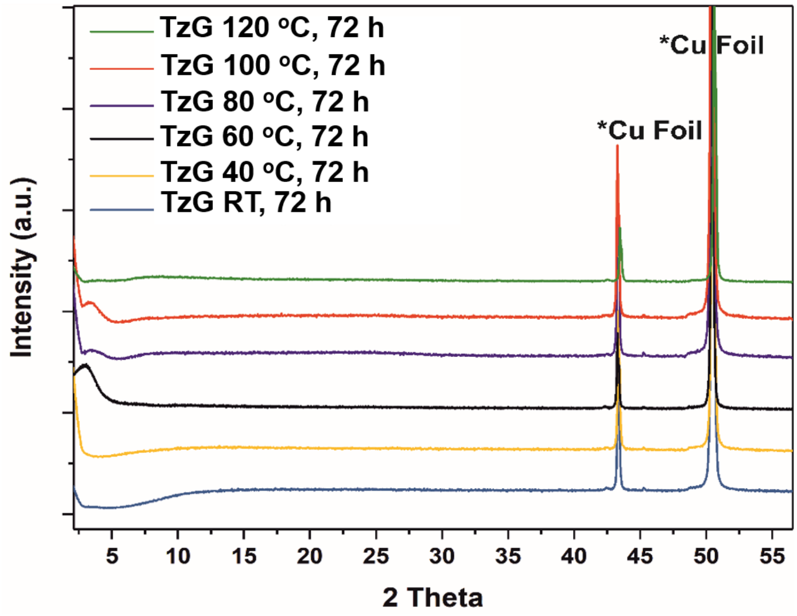

Fig. S10 Effect of temperature on PXRD profiles of TzG, all the PXRD measurements were performed on as-synthesized TzG samples on copper foil.

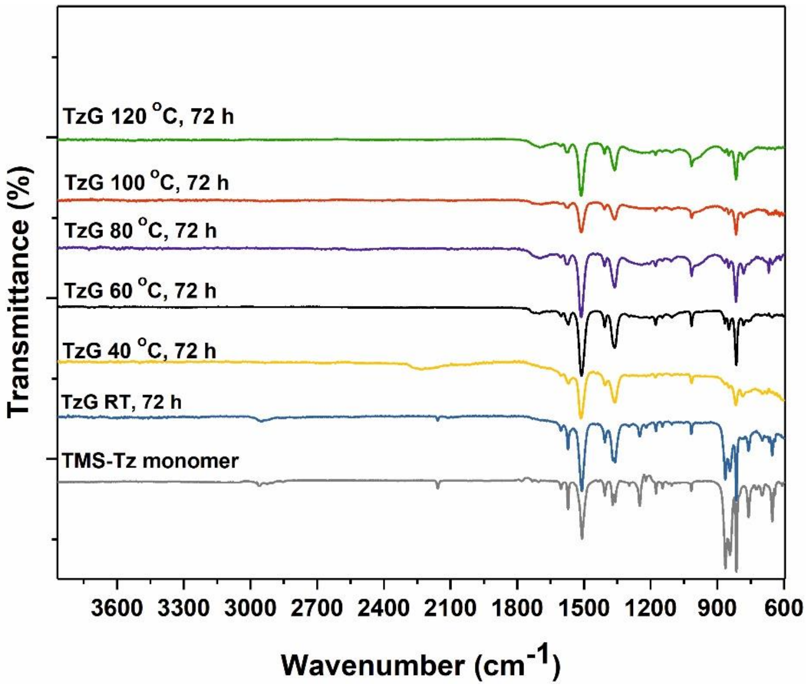

Fig. S11 FTIR measurements of TzG synthesized at different temperatures.

Optimized protocol for Synthesis of TzG:

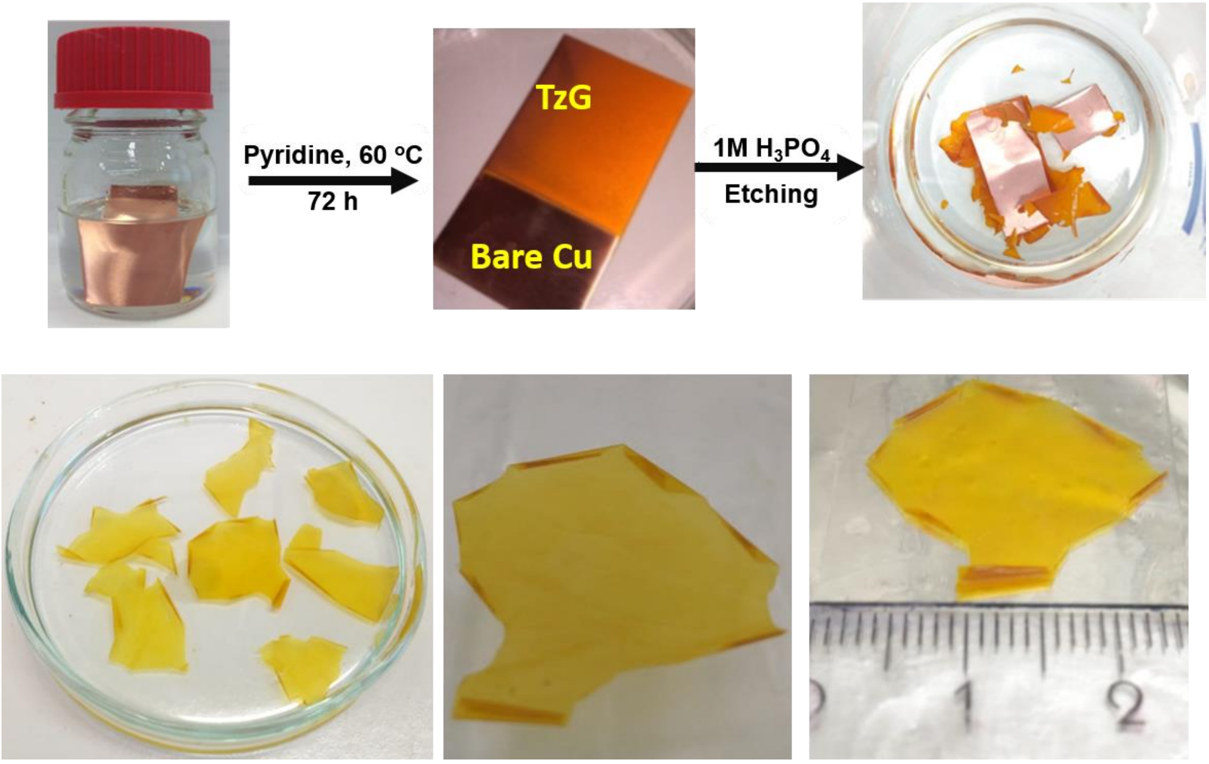

Fig. S12 Synthesis of TzG on a copper surface and etching protocol used for isolating films.

In a Schott Duran bottle pyridine (50 mL) and 2,4,6-tris(4-[(trimethylsilyl)ethynyl]phenyl)-1,3,5-triazine TMS-Tz **(3)** (50 mg, 0.08 mmol) were added. It is important to note that the TMS-Tz monomer is not readily soluble in pyridine, and was dissolved using ultrasonication. Then the reaction vessel was charged with a copper foil (as received). The reaction vessel was then sealed tightly with a teflon cap and heated in an oven at 60 ^{o}C for 72 h. After the reaction, the copper foil was removed from the reaction mixture and washed with DMF, THF, $CHCl_3$, deionized water, and MeOH. Then polymer-coated copper foil was submerged in an aqueous solution of H_3PO_4 (1M). Within seconds, the flakes delaminate from the copper substrate (see Supplementary video V1). The as-synthesized TzG were then subjected to a wash with HCl (1M) to remove excess copper, followed by washing with NH_4OH solution (1M) to remove the trapped HCl salts. The films were then subjected to wash with THF, MeOH, DMF, and Acetone. Finally, the product was dried under vacuum for 24 h at 120 °C to yield yellow-orange films.

Table. S1: Combustion elemental analysis (EA) data for TzG after drying

Sample Name		C (wt%)	H (wt%)	N (wt%)
**TzG[a]**	Theoretical	85.73	3.17	11.11
	Experimental	74.52	3.43	10.42
TzG[b]	Experimental	83.50	4.04	10.44

a: sample dried at 120 °C for 12 h under vacuum

b: sample dried at 180 °C for 72 h under vacuum

Thermogravimetric analysis (TGA):

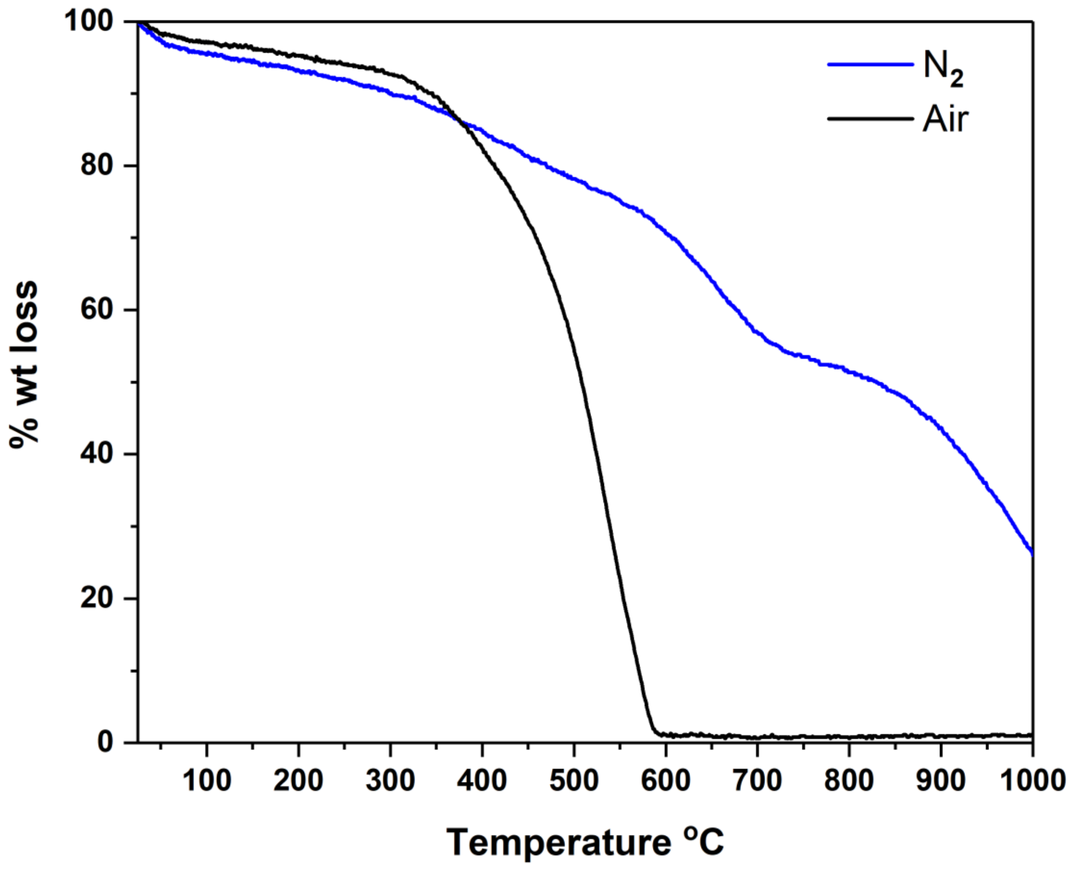

Fig. S13 Thermogravimetric analysis of TzG under air and nitrogen environments residual mass of 0.6% and 25% under air and nitrogen, respectively.

X-ray photoelectron spectroscopy (XPS):

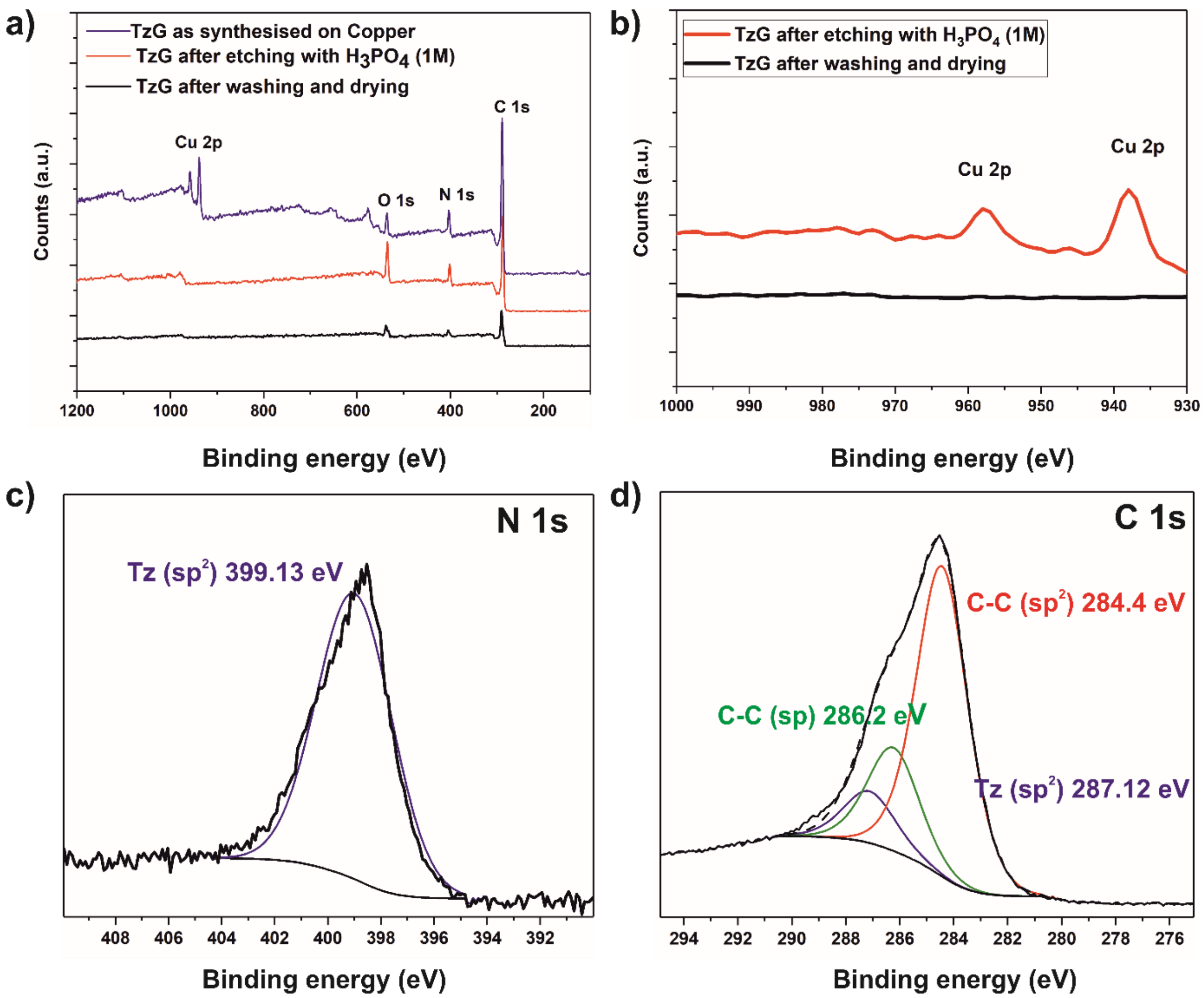

Fig. S14 XPS data of TzG a) Comparison between the samples at different stages, b) XPS of Cu 2p region before and after washing c) corresponds to N 1s, and d) corresponds C 1s environments respectively.

Energy-dispersive X-ray (EDX):

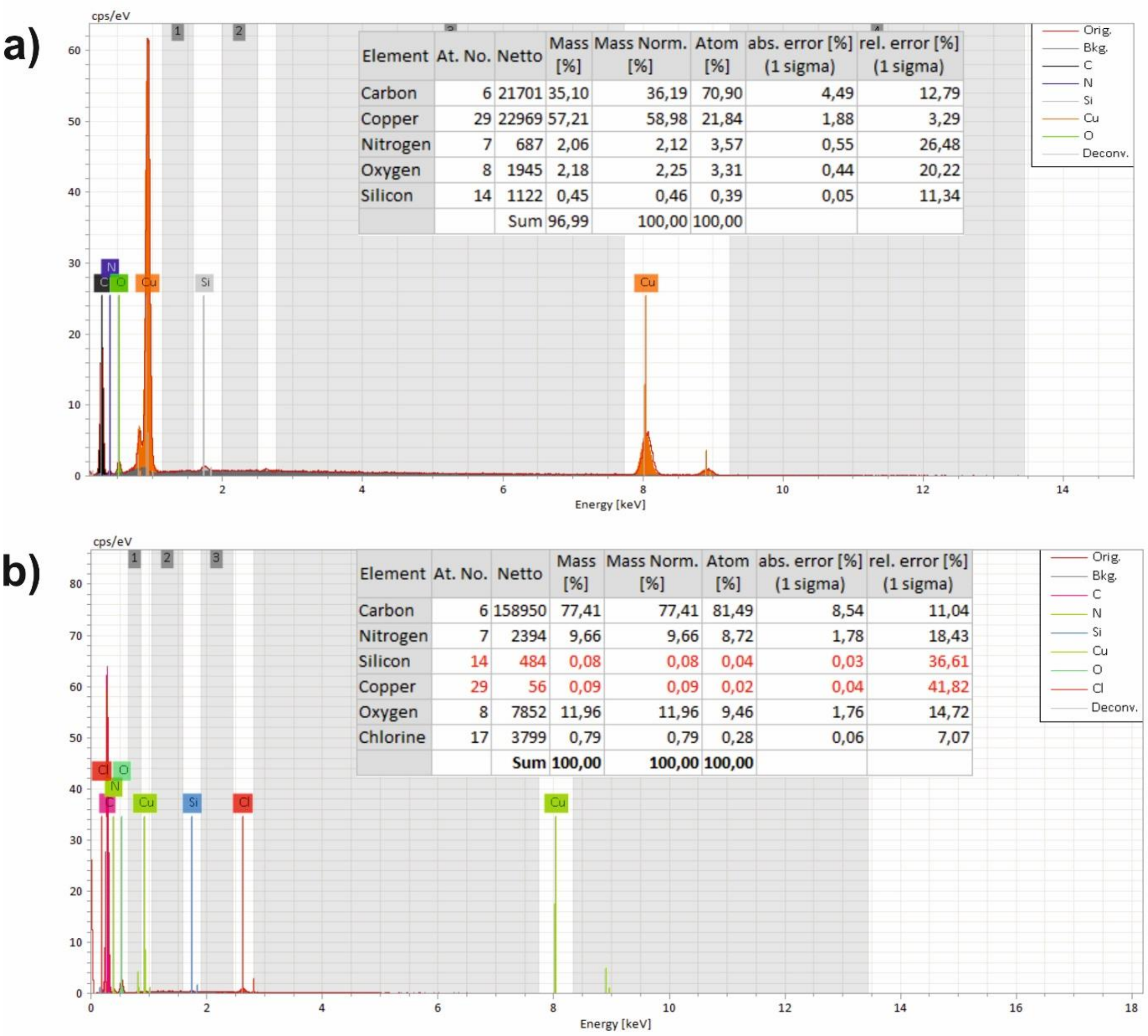

Element	At. No.	Netto	Mass [%]	Mass Norm. [%]	Atom [%]	abs. error [%] (1 sigma)	rel. error [%] (1 sigma)
Carbon	6	21701	35,10	36,19	70,90	4,49	12,79
Copper	29	22969	57,21	58,98	21,84	1,88	3,29
Nitrogen	7	687	2,06	2,12	3,57	0,55	26,48
Oxygen	8	1945	2,18	2,25	3,31	0,44	20,22
Silicon	14	1122	0,45	0,46	0,39	0,05	11,34
		Sum	96,99	100,00	100,00		

Element	At. No.	Netto	Mass [%]	Mass Norm. [%]	Atom [%]	abs. error [%] (1 sigma)	rel. error [%] (1 sigma)
Carbon	6	158950	77,41	77,41	81,49	8,54	11,04
Nitrogen	7	2394	9,66	9,66	8,72	1,78	18,43
Silicon	14	484	0,08	0,08	0,04	0,03	36,61
Copper	29	56	0,09	0,09	0,02	0,04	41,82
Oxygen	8	7852	11,96	11,96	9,46	1,76	14,72
Chlorine	17	3799	0,79	0,79	0,28	0,06	7,07
		**Sum**	**100,00**	**100,00**	**100,00**		

Fig. S15 Energy-dispersive X-ray (EDX) spectra and the ratio of elements detected by EDX in wt% of (a) TzG as synthesized on copper, and (b) TzG after washing and drying.

Fourier transformed infrared spectroscopy (FT-IR):

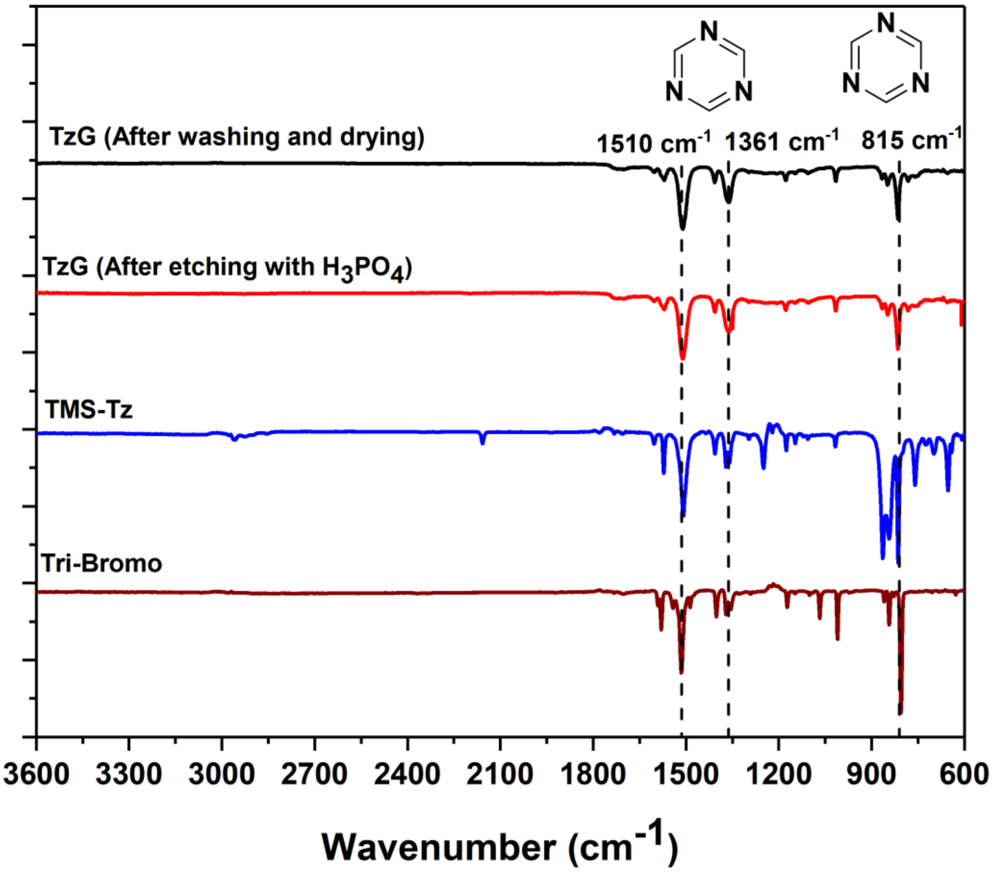

Fig. S16 FT-IR spectra of monomer 1, (2,4,6-tris(4-bromophenyl)-1,3,5-triazine) (maroon), monomer, 2,4,6-tris(4-[(trimethylsilyl)ethynyl]phenyl)-1,3,5-triazine (TMS-Tz) (blue), TzG (red) after etching with H_3PO_4 and TzG after washing and drying (black).

Solid-state Raman of TzG after drying:

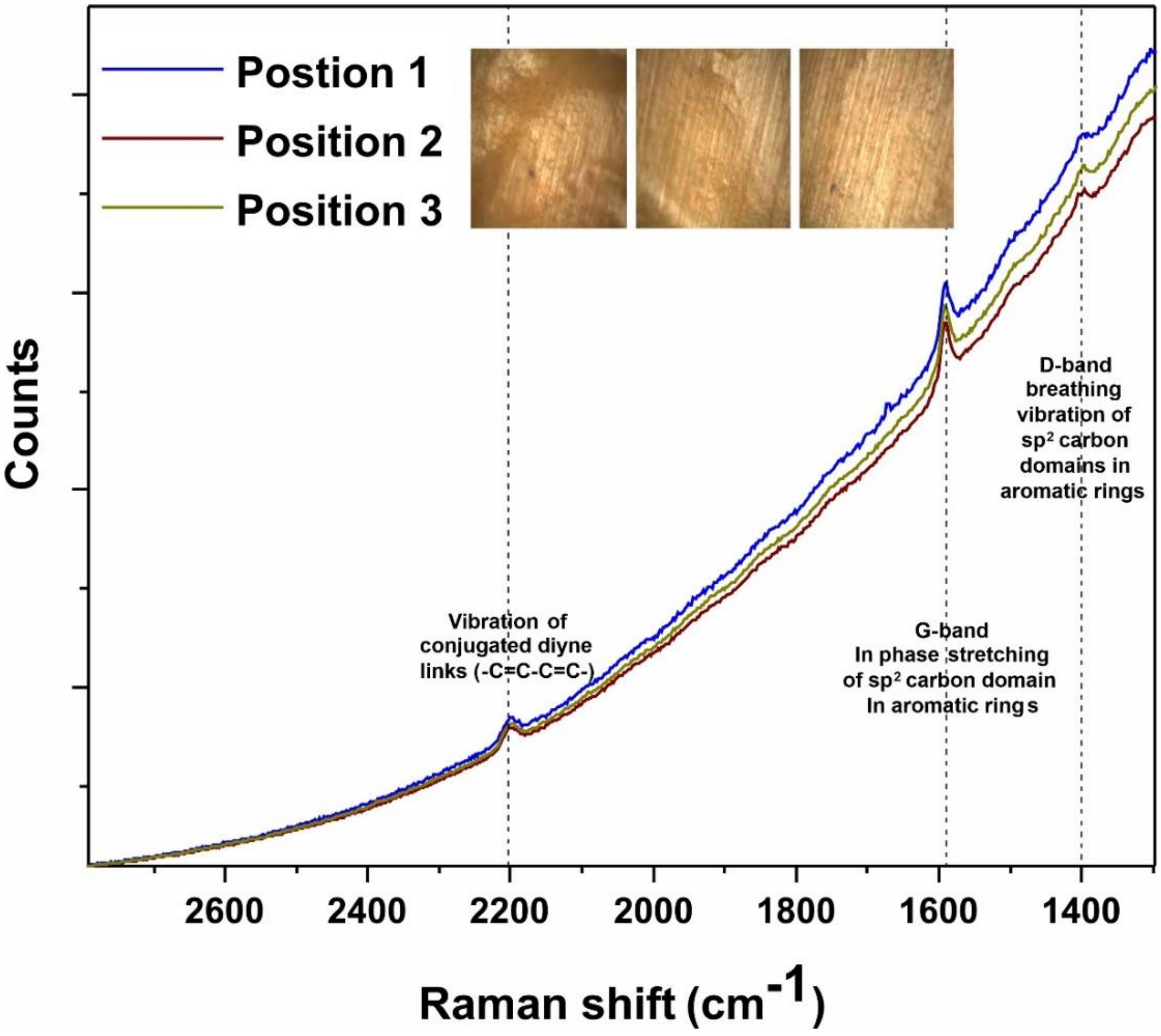

Fig. S17 Solid-state Raman measurement of TzG at different positions, inset optical images of different positions.[3]

DFT model structures used for PXRD assignment:

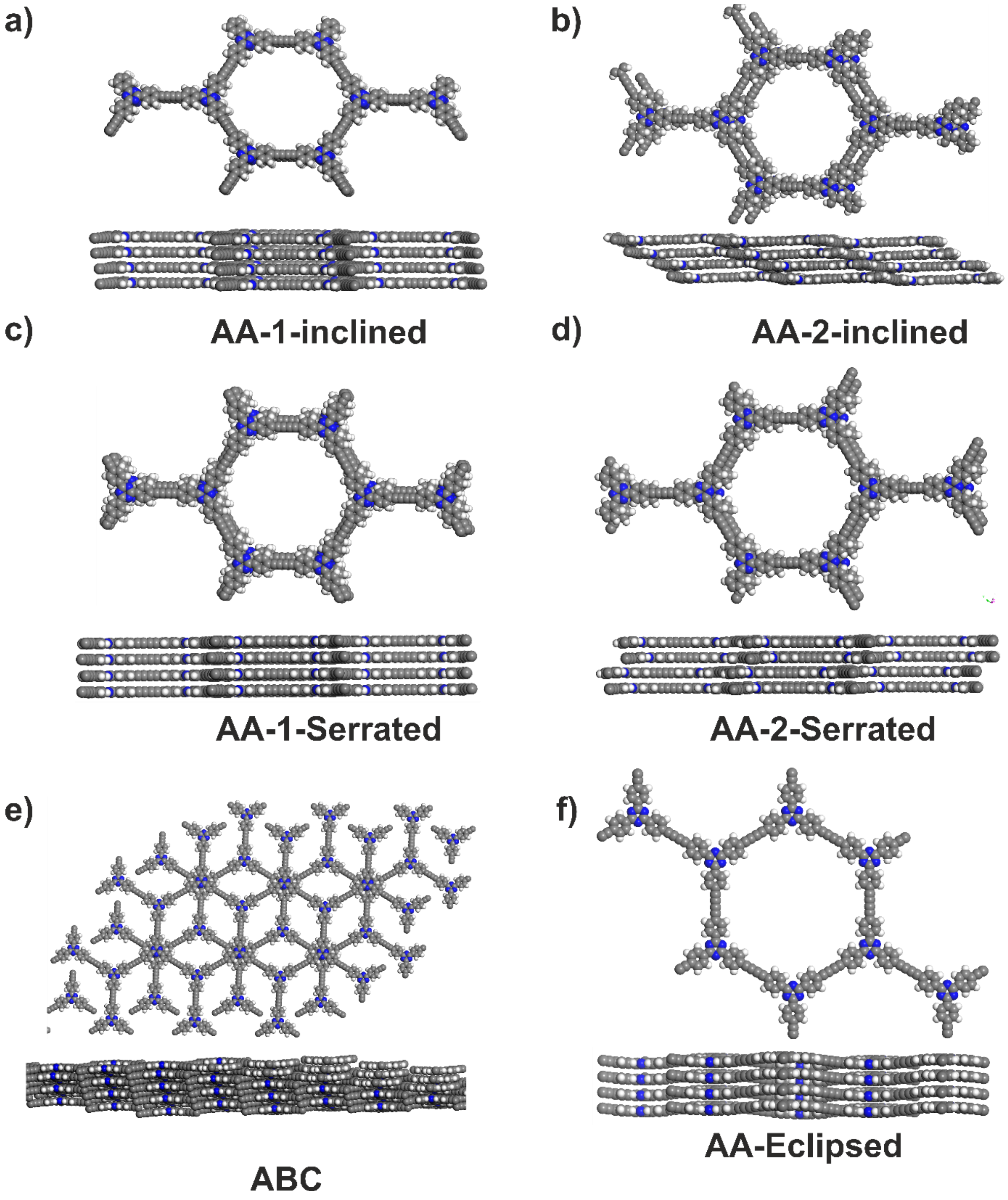

Fig. S18 DFT model structures used for PXRD assignment a) AA-1-inclined, b) AA-2-inclined, c) AA-1-serrated, d) AA-2-serrated, e) ABC stack and f) AA-Eclipsed.

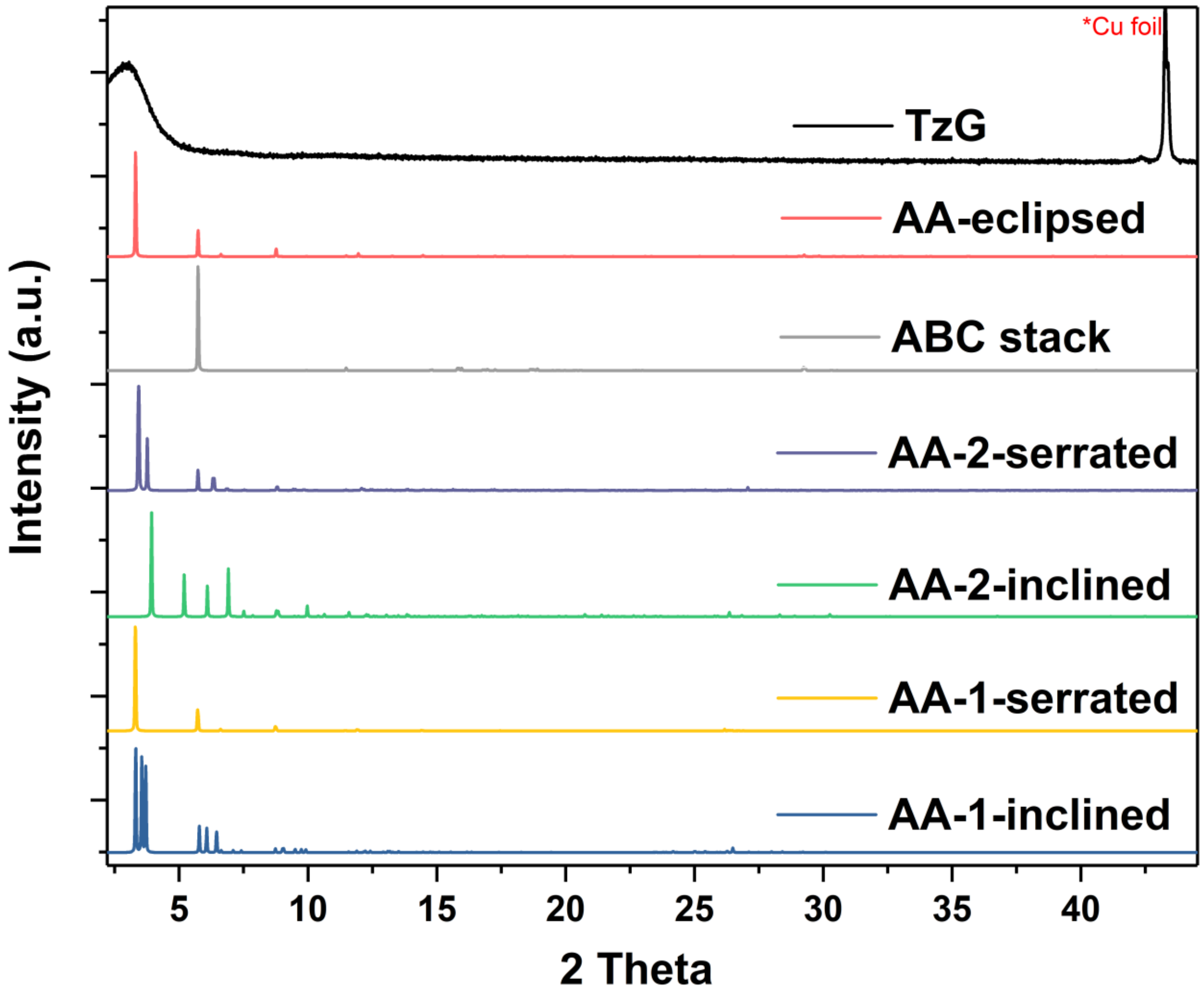

Fig. S19 Comparison of PXRD pattern of TzG (black) synthesised at 60 ºC and corresponding simulated patterns for AA-1-inclined, AA-1-serrated, AA-2-inclined, AA-2-serrated, ABC, and AA-eclipsed stack.

Scanning electron microscope (SEM):

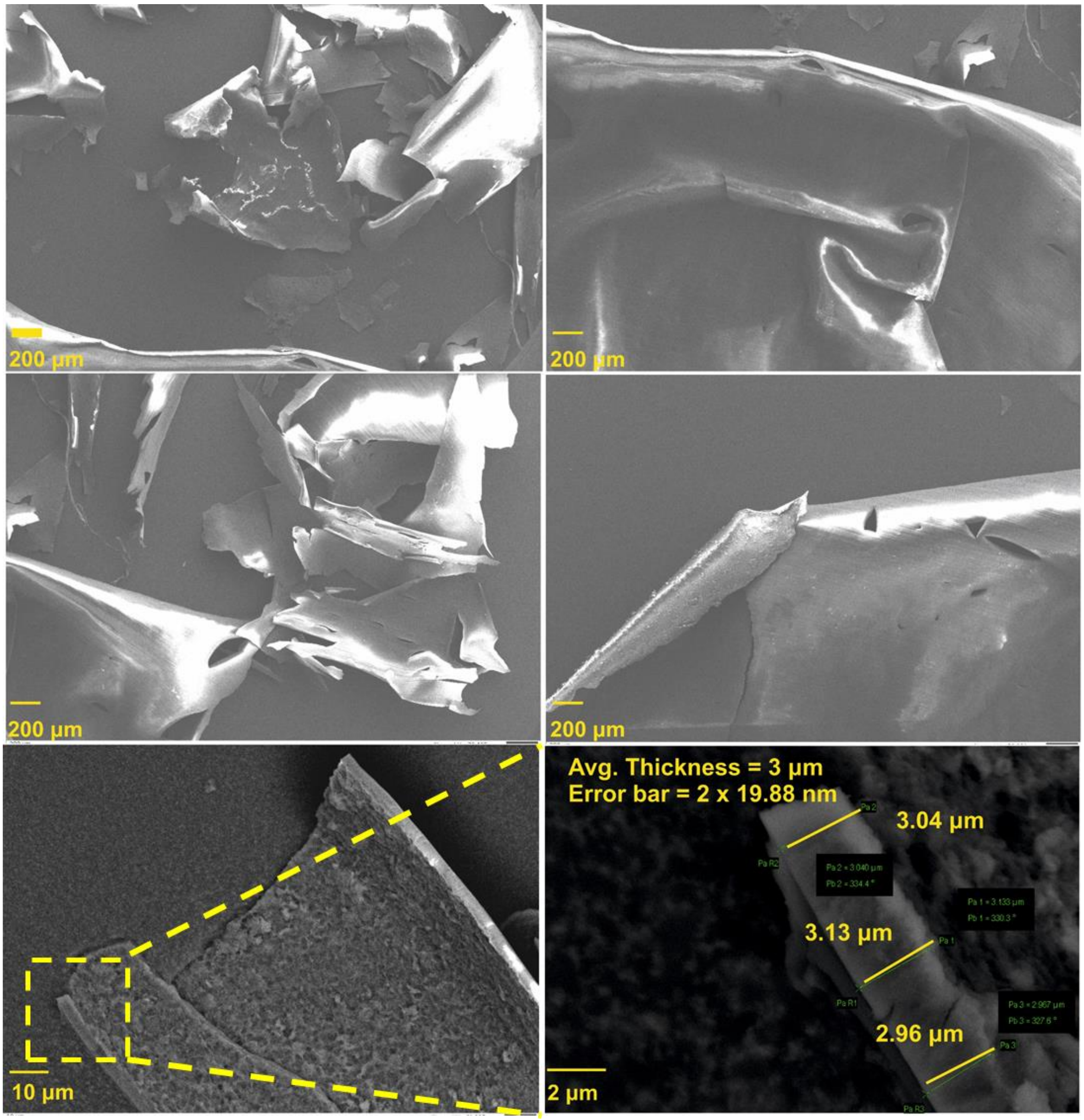

Figure S20 SEM images of TzG s after washing and drying show layered structure with contours from the copper foil and the thickness measurement. Note the imprints of the copper foil in the form of striations can be clearly seen on the flakes.

Transmission electron microscopy (TEM)

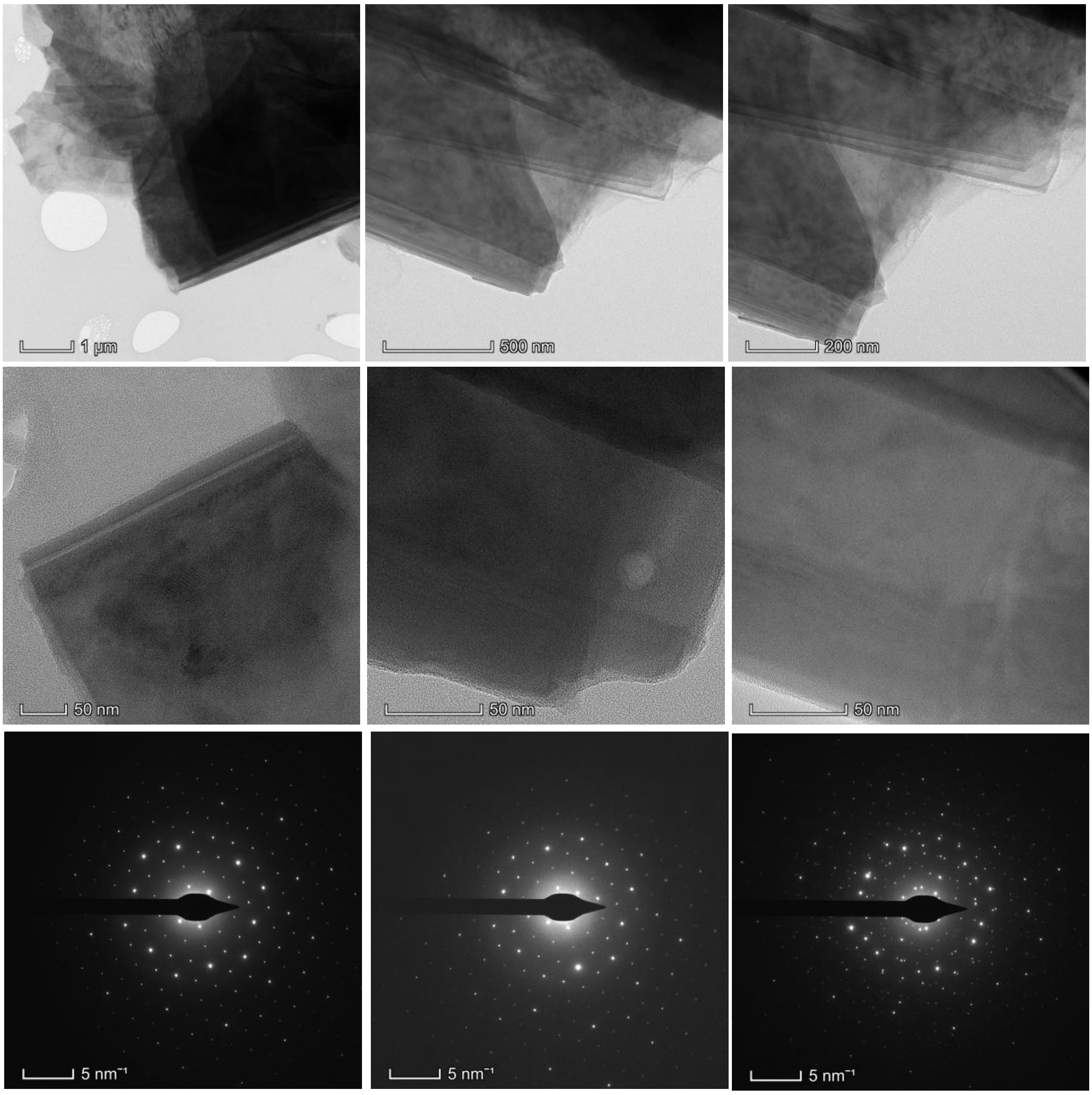

Fig. S21 HR-TEM/SAED images of TzG after drying.

SAED comparison with AA stacking modes

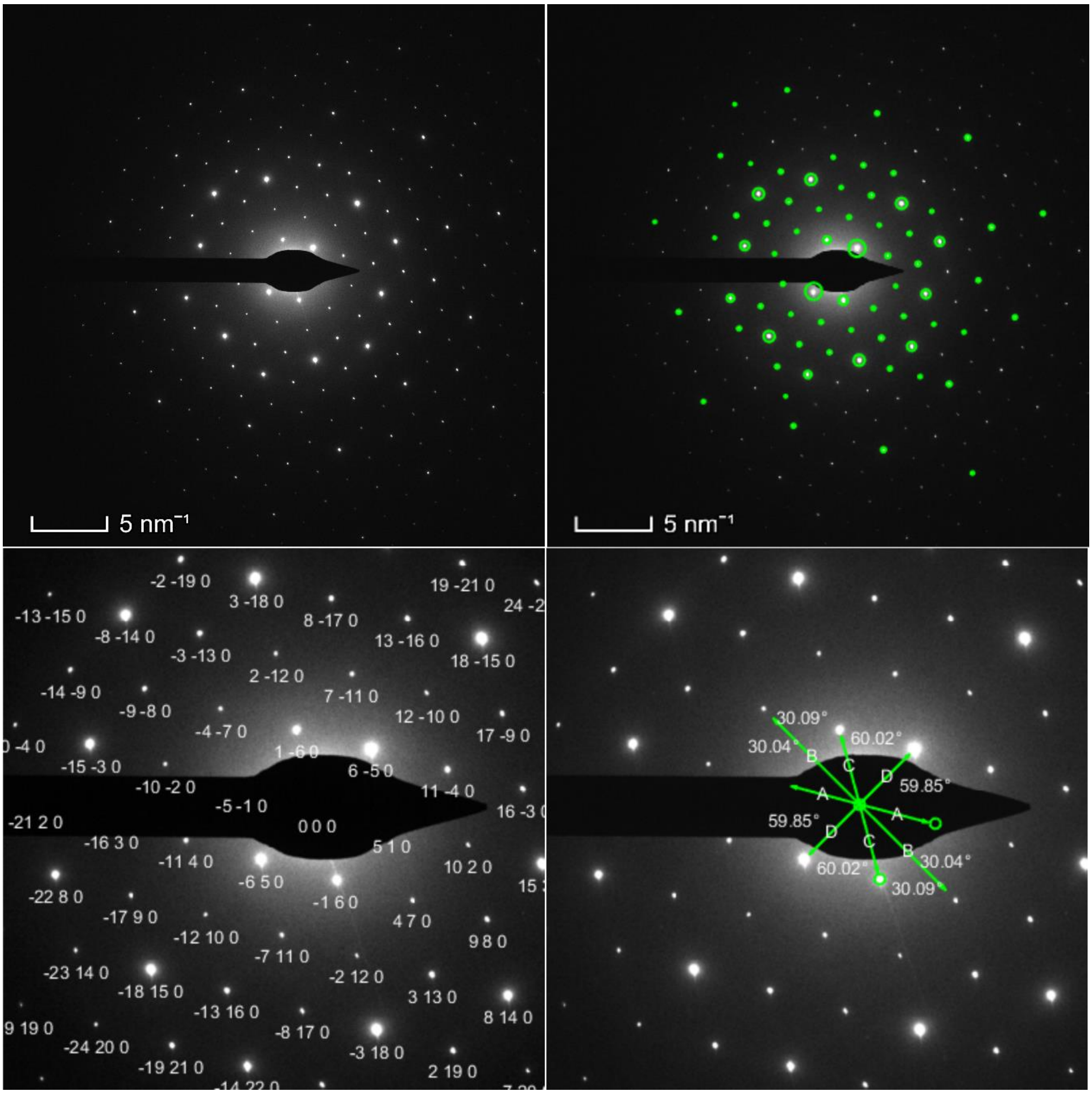

Fig. S22 Structure analysis of crystalline TzG, the best fit is achieved by comparing the experimental SAED patterns with the optimized, AA-Eclipsed TzG structure using CystTBox.

Solid-state UV-Vis measurements:

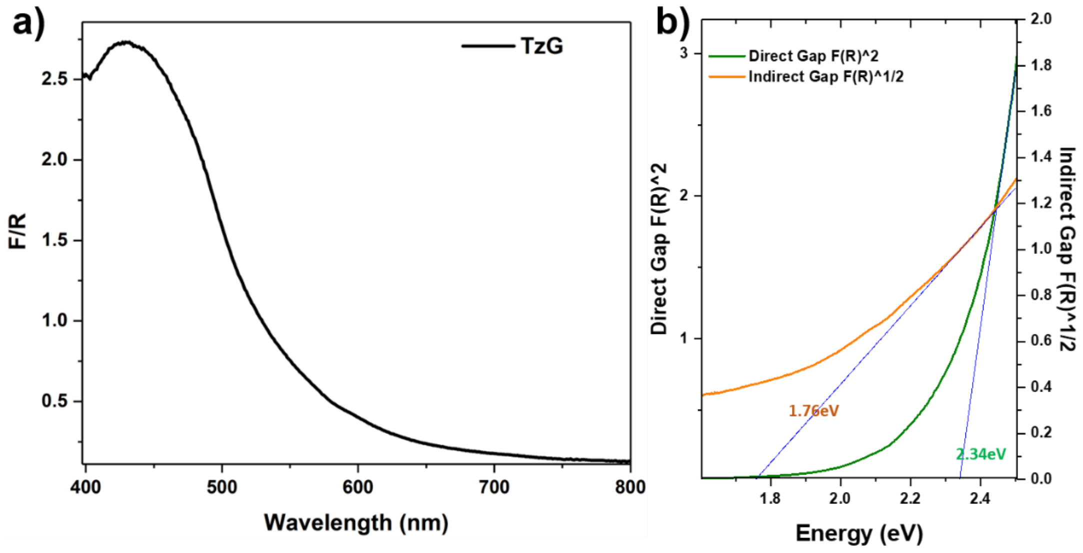

Fig. S23 a) Solid-state UV-Vis of TzG after drying and b) Kubelka-Munk plots for TzG assuming direct (dark) and indirect (light) bandgap.

Solid-state photoluminescence emission spectra:

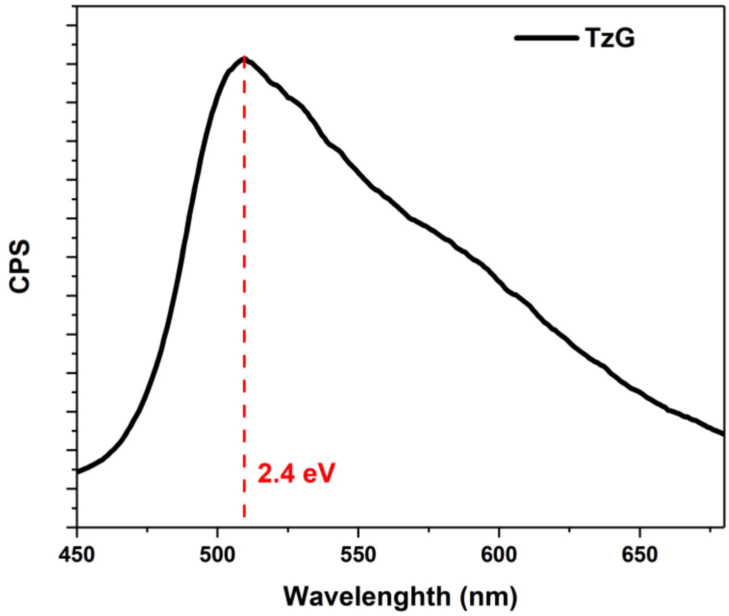

Fig. S24 a) Solid-state photoluminescence emission spectra of TzG flake after drying, excitation wavelength 400 nm.

Effect of dissolved oxygen on coupling efficiency

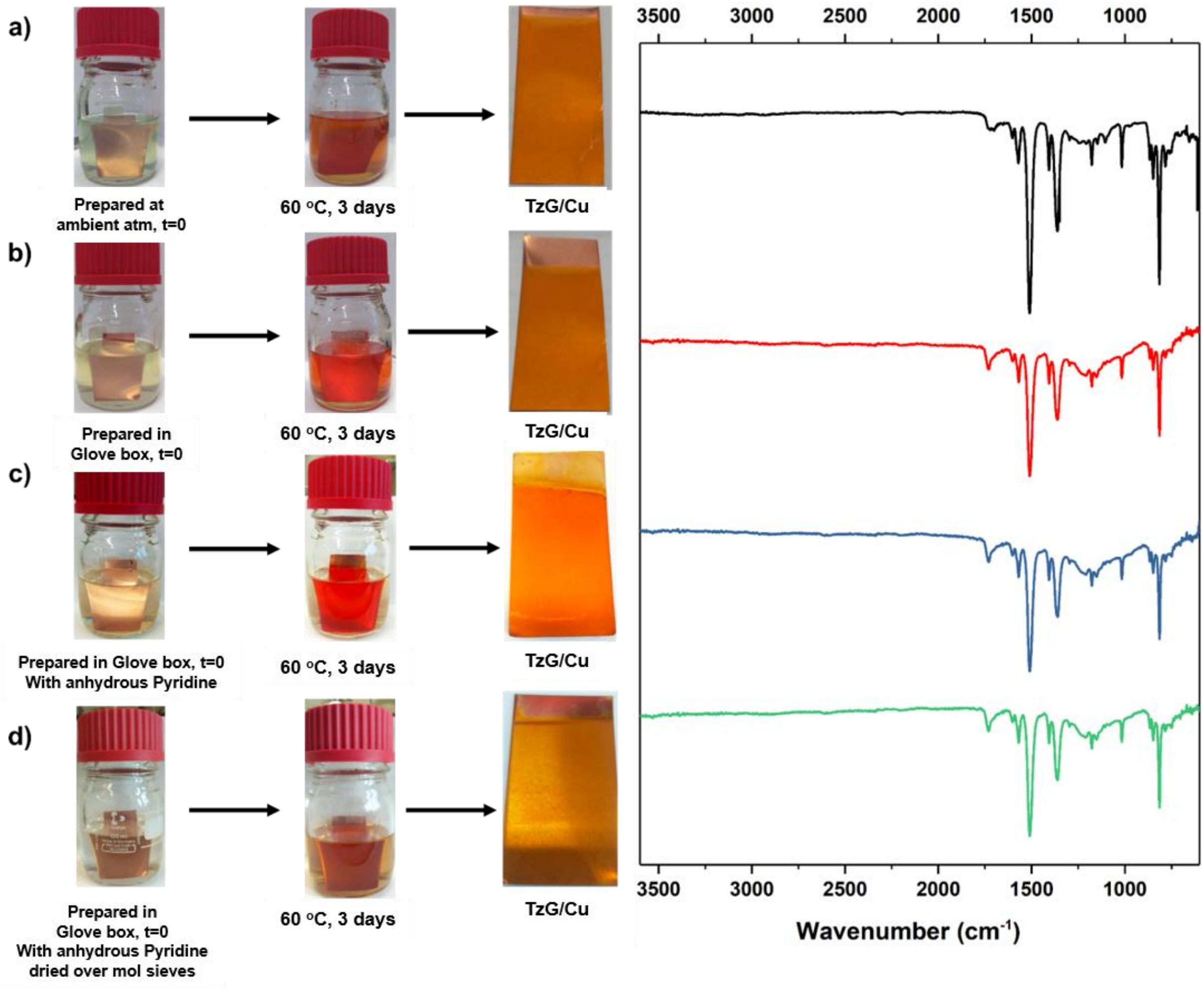

Fig. S25 Comparative study of TzG growth on copper foil, to study the effect of dissolved oxygen on coupling efficiency a) in the glove box, b) at ambient atmosphere, c) in the glove box with dried pyridine and d) with anhydrous pyridine dried and degassed.

XPS analysis of as-received vs Argon sputtered Copper foil

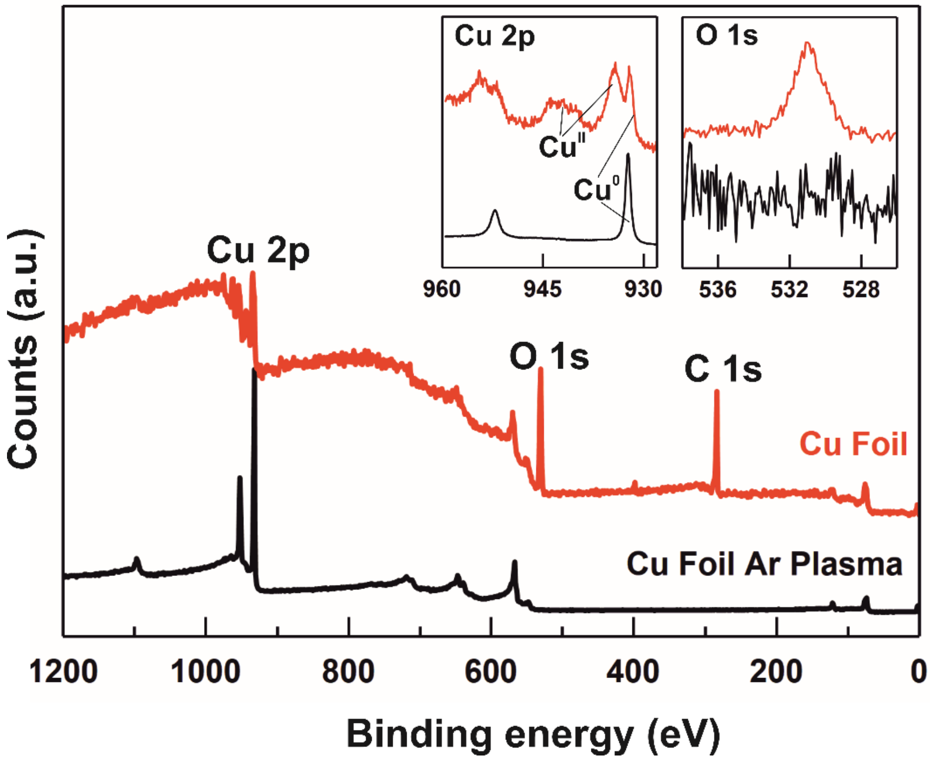

Fig. S26 X-ray photoelectron spectroscopy (XPS) data for as-received copper foil (red) and Ar sputtered copper foil (black).

Control experiments with trimethylsilylethynylbenzene with $Cu(OH)_2$, CuO

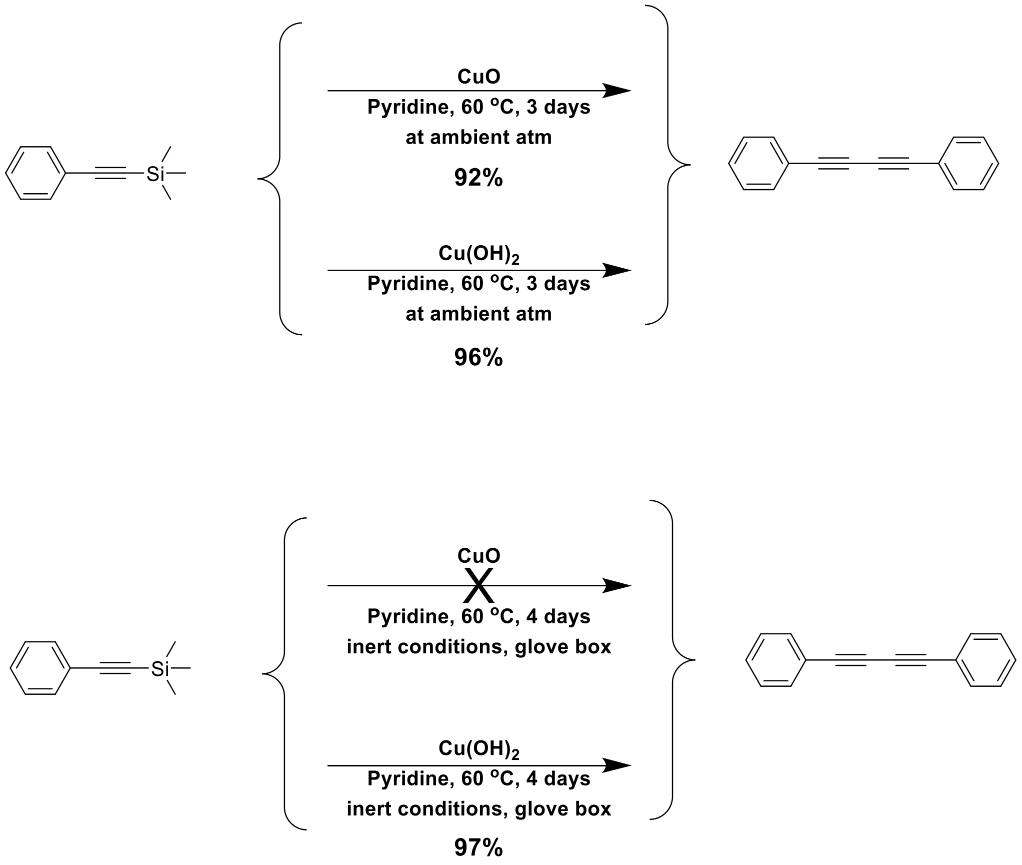

Fig. S26 Control experiments to verify active species for the formation of diacetylene bridge, reaction conditions: (1 mmol) of TMS-Acetylene, (3 mmol) of copper salts, 60 °C, 3 days; note: For the glove box experiments, copper salts were dried at 120 °C for 12 h, and anhydrous pyridine was degassed with argon for 1 h before use.

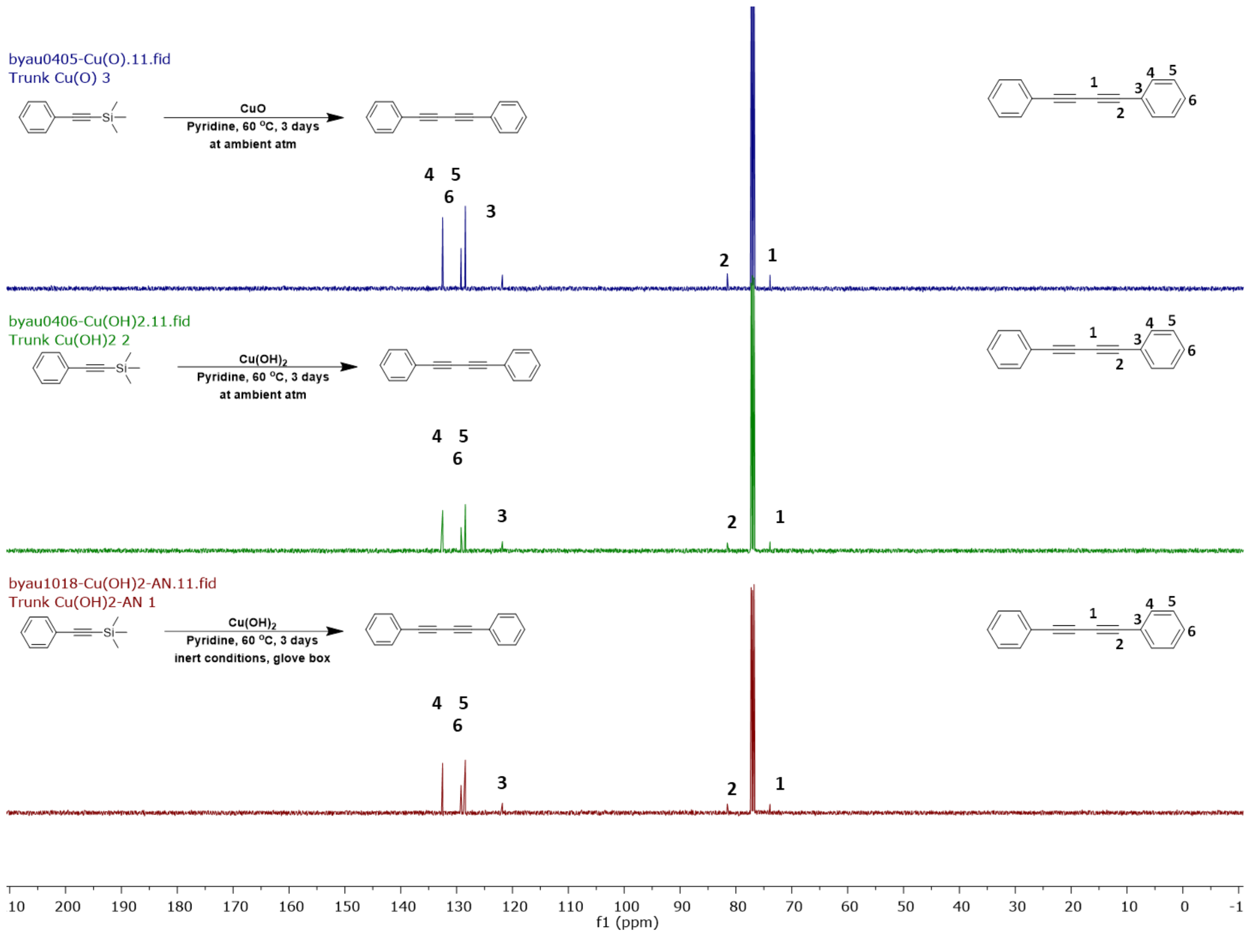

Fig. S27 ^{13}C NMR of the products isolated from the above control experiments, reaction conditions: (1 mmol) of TMS-Acetylene, (3 mmol) of copper salts, 60 ^{o}C, 3 days; note: For the glove box experiments, copper salts were dried at 120 ^{o}C for 12 h, and anhydrous pyridine was degassed with argon for 1 h before use, the product was isolated by pouring the reaction mixture over ice.

Control experiments using TMS-Tz with $Cu(OH)_2$, CuO

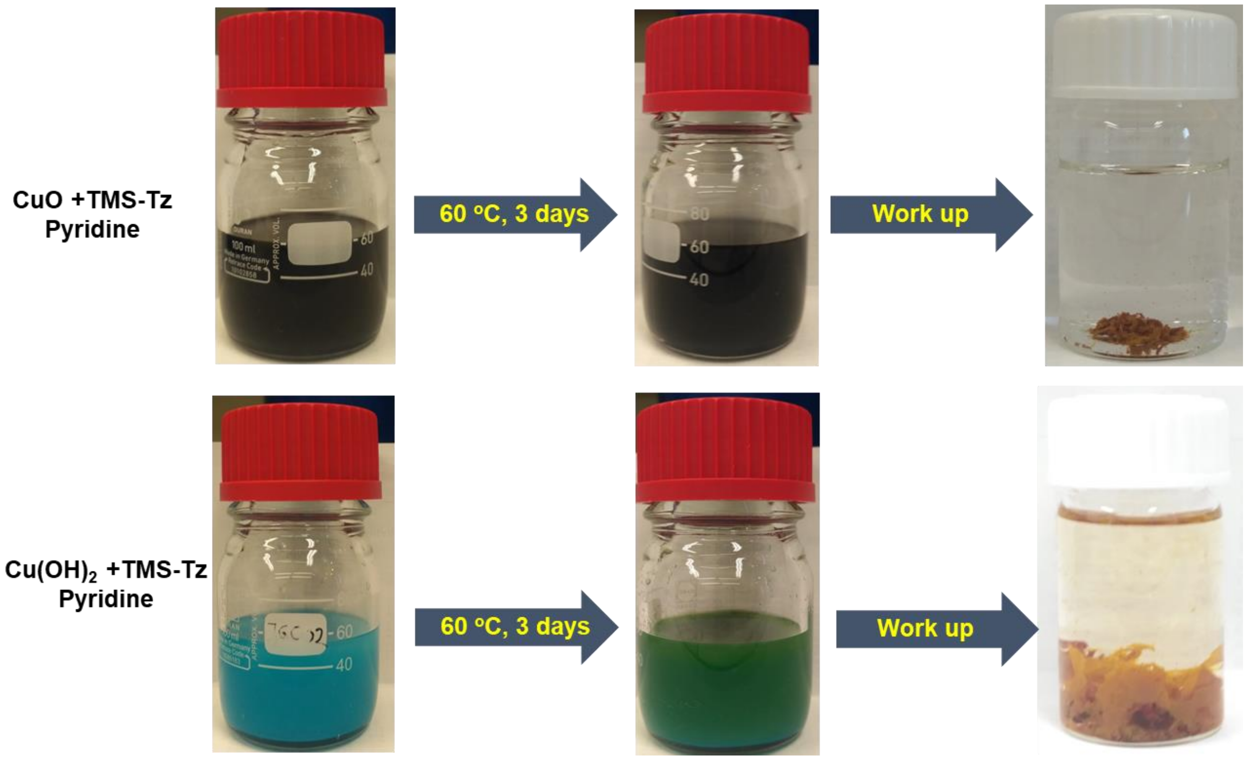

Fig. S28 Polymerization trials using CuO, and $Cu(OH)_2$, reaction conditions: (0.08 mmol) of TMS-Tz and (1.2 mmol) of copper salts, in 50 mL pyridine heated at 60 °C, 3 days.

Wagner Plot and Auger parameters

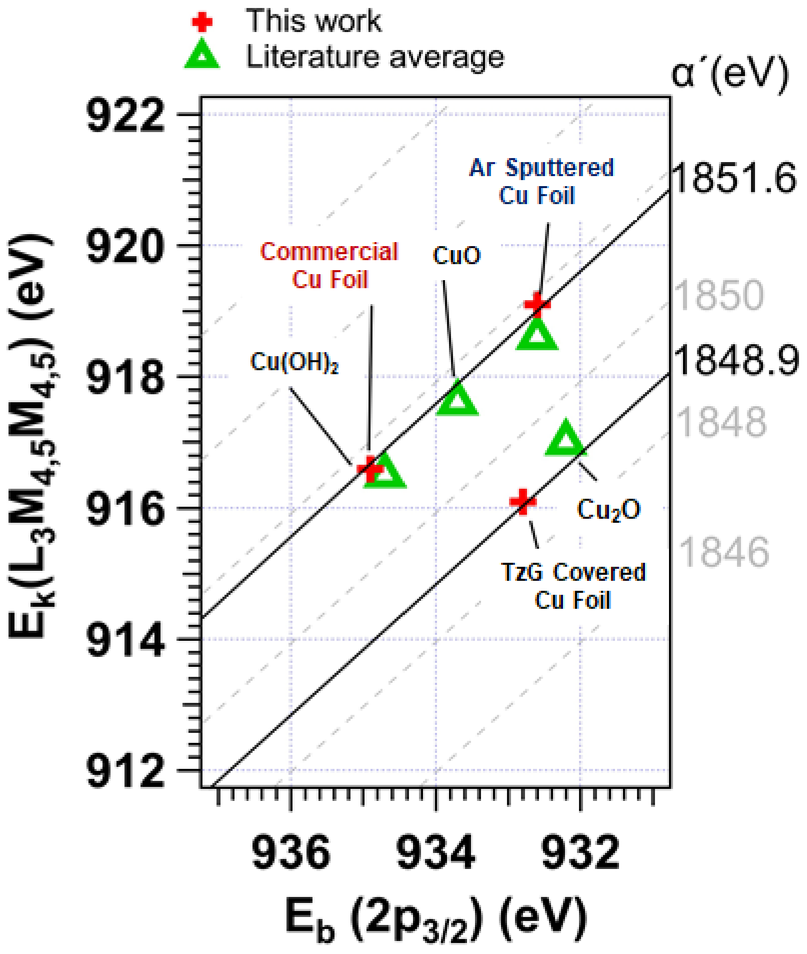

Fig. S30 Cu $2p_{3/2}$–Cu $L_3M_{4,5}M_{4,5}$ Wagner (chemical state) plot including literature [4, 5], and present work. Constant Auger parameter lines are also plotted together with their corresponding α´ values on the right side of the graph. The black lines correspond to the Auger parameters determined in this work.

References:

1. D. Schwarz, Y. Noda, J. Klouda, K. Schwarzová-Pecková, J. Tarábek, J. Rybáček, J. Janoušek, F. Simon, M. V. Opanasenko and J. Čejka, *Advanced Materials*, 2017, **29**, 1703399.
2. D. Schwarz, A. Acharjya, A. Ichangi, Y. S. Kochergin, P. Lyu, M. V. Opanasenko, J. Tarábek, J. Vacek Chocholoušová, J. Vacek and J. Schmidt, *ChemSusChem*, 2019, **12**, 194-199.
3. G. Li, Y. Li, H. Liu, Y. Guo, Y. Li and D. Zhu, *Chemical Communications*, 2010, **46**, 3256-3258.
4. M. C. Biesinger, *Surface and Interface Analysis*, 2017, **49**, 1325-1334.
5. G. Moretti, A. Palma, E. Paparazzo and M. Satta, *Surface Science*, 2016, **646**, 298-305.

Author contributions:

All authors have actively contributed to the work presented in this paper R.K. and M.J.B. conceived the research project. R.K. planned and carried out the synthetic experiments, analysed the data and wrote the paper. J.H. carried out the XPS measurements and analysis. M.T. carried out the mechanistic studies and assisted in manuscript writing. D.B. carried out the argon plasma experiments and analysis. J.M. carried out the SEM and SEM-EDX measurements. P.A. performed the XPS measurements and wagner plots. A.M. helped with the glove box experiments. M.D. performed the solid-state NMR experiments. D.K optimized the GC method and did the GCMS measurements and M.J.B. conceived the experiments helped in correcting the paper.
